# Potent Bidirectional Cross-Talk Between Plasmacytoid Dendritic Cells and γδT Cells Through BTN3A, Type I/II IFNs and Immune Checkpoints

**DOI:** 10.3389/fimmu.2020.00861

**Published:** 2020-05-06

**Authors:** Pauline Girard, Benedicte Ponsard, Julie Charles, Laurence Chaperot, Caroline Aspord

**Affiliations:** ^1^Etablissement Français du Sang Auvergne Rhone-Alpes, Research and Development Laboratory, Grenoble, France; ^2^Université Grenoble Alpes, INSERM, CNRS, Team Immunobiology and Immunotherapy in Chronic Diseases, Institute for Advanced Biosciences, Grenoble, France; ^3^Dermatology Department, Grenoble Alpes University Hospital, Grenoble, France

**Keywords:** pDCs, γδ T cells, cross-talk, immune checkpoint, BTN3A

## Abstract

Plasmacytoid DCs (pDCs) and γδT cells are both critical players in immunosurveillance against pathogens and cancer due to their ability to sense microbes and cell stress through recognition of pathogen-associated molecular patterns or altered metabolism [phosphoantigens (PAgs)]. Their unique features, high functional plasticity and ability to interact with many immune cell types allow them to bridge innate and adaptive immunity, initiating and orientating widely immune responses, hence contributing to protective and pathogenic immune responses. Yet, despite strategic and closed missions, potential interactions between pDCs and γδT cells are still unknown. Here we investigated whether there is interplay between pDCs and γδT cells and the underlying molecular mechanisms. Purified human pDCs and γδT cells were cocultured in presence of TLR-L, PAg, and zoledronate (Zol) to mimic both infectious and tumor settings. We demonstrated that TLR7/9L- or Zol-stimulated pDCs drive potent γδT-cell activation, Th1 cytokine secretion and cytotoxic activity. Conversely PAg-activated γδT cells trigger pDC phenotypic changes and functional activities. We provided evidence that pDCs and γδT cells cross-regulate each other through soluble factors and cell-cell contacts, especially type I/II IFNs and BTN3A. Such interplay could be modulated by blocking selective immune checkpoints. Our study highlighted crucial bidirectional interactions between these key potent immune players. The exploitation of pDC-γδT cells interplay represents a promising opportunity to design novel immunotherapeutic strategies and restore appropriate immune responses in cancers, infections and autoimmune diseases.

## Introduction

Plasmacytoid DCs (pDCs) and γδ T cells are both critical players in immunosurveillance against pathogens and cancer and orchestrate immune responses (1, 2). These potent sentinels sense microbes and cell stress through recognition of pathogen-associated molecular patterns (PAMPs) or altered metabolism in a complementary manner: pDCs can directly recognize pathogenic motifs through Toll-like receptors (TLR), while γδ T cells sense stress-induced antigens such as phosphoantigens (PAgs) on transformed or infected cells via their T-cell receptor (TCR). They exhibit a rapid response through, respectively, massive type I or type II IFN secretion and subsequently initiate immune responses. Their unique features, high functional plasticity and ability to interact with many other immune cell types allow them to bridge innate and adaptive immunity, triggering efficient immune responses against pathogens and cancer. pDCs and γδ T cells owe essential contribution to many types of protective immune responses but also in immunopathology (3, 4). Notably, pDCs (4, 5) and γδ T cells (6–9) have been both involved positively or negatively in cancer, infections and autoimmunity: they have been shown to display pro- and anti-tumor effects, to be potent effectors against pathogens (especially viruses and bacteria), and to undergo hyperactivation in autoimmune and chronic inflammatory diseases. Both pDCs and γδ T cells are exploited as vector or target for immunotherapy of cancers and infectious diseases (10, 11). Yet, despite strategic and closed missions, potential interactions between pDCs and γδ T cells are still unknown.

pDCs play a crucial role in the initiation and orientation of anti-viral and anti-tumor immune responses and are major players in the regulation of immunity (1, 4, 5, 12, 13). The expression of TLR7 and 9 confers on them the ability to recognize pathogenic motifs (single stranded RNA, unmethylated CpG-containing DNA). Upon activation, pDCs exhibit robust IFN-α production and promote innate and adaptive immune responses. The functional plasticity of pDCs as well as their ability to interact with different immune cells allows them to direct immunity toward multiple profiles (immunity or tolerance) according to the microenvironment (5). pDCs promote antiviral responses and have been implicated in the pathogenesis of autoimmune diseases (12). pDCs also elicit anti-tumor responses by their ability to induce antigen-specific adaptive responses (14, 15) or by exerting a direct cytotoxic activity toward the tumor cells via TRAIL (16, 17), but are found to be subverted in many cancers and to contribute to the establishment of an immunosuppressive tumor microenvironment (18, 19).

γδ T cells are unconventional T cells playing a major role in immune responses against various microbes (parasites, bacteria, viruses), stressed cells and tumor cells (3, 8, 20). γδ T cells are crucial effectors in immunosurveillance of tumor (10, 21) and infections (2, 3, 8) due to their prompt activation, their capacity to recognize tumor- and stress-associated ligands neglected by conventional αβT cells in an MHC-unrestricted manner, their potential to kill transformed and infected cells through secretion of cytotoxic pro-apoptotic protease granzymes and pore-forming molecules perforin, and their ability to secrete immunostimulatory cytokines in particular IFNγ and TNFα regulating and potentiating the effectiveness of other immune cells leading to coordinated immune responses (20). γδ T cells can be subdivided based on their δ TCR chain. Tissue-associated γδ T cells harbor mainly the Vδ1 TCR and recognize stress-related antigens, whereas the majority of circulating γδ T cells bears the Vδ2 TCR. γδ T cells bearing the TCR Vγ9Vδ2 recognize unprocessed non-peptide molecules, PAg, derived from the isoprenoid/mevalonate or non-mevalonate pathways such as (E)-4-hydroxy-3-methyl-but-2-enyl pyrophosphate (HMB-PP) produced by many pathogens, and isopentenyl pyrophosphate (IPP), which accumulate intracellularly during dysregulated metabolism in many tumors. Cells treated with aminobiphosphonates (ABP) such as Zol are potent activators of Vγ9Vδ2 T cells due to the accumulation of IPP upon inhibition of the IPP metabolizing farnesyl diphosphate synthase (FPPS). The activation of Vγ9Vδ2 T cells by PAg requires the contact with cells expressing CD277/butyrophilin-3 A1 (BTN3A1) molecule. Two major hypotheses explain PAg-mediated activation of Vγ9Vδ2 T cells (22). The “allosteric model,” which is supported by recent literature, suggests that PAg-binding to the intracellular domain of BTN3A1 provokes conformational changes in its extracellular domain, allowing the binding of BTN3A1 to Vγ9Vδ2 TCR and subsequent activation (23–27). The “antigen-presenting model” proposes that upon export in the extracellular microenvironment, PAg presented by BTN3A1 form BTN3A1-PAg complexes that directly bind the Vγ9Vδ2 TCR (28). Beside sensing the dysregulation of intracellular PAg levels, γδ T cells also recognize induced self-ligands such as stress-inducible MICA/MICB molecules, upregulated at the surface of stressed or tumor cells (29) through NKG2D (30). γδ T cells hence display a broad reactivity against tumors and pathogens by sensing microbial infections and metabolic changes found in transformed, infected, or drug-treated cells.

Cross-talks between DCs and γδ T cells are still not fully characterized. Bidirectional interactions between monocyte-derived DCs (moDCs) and γδ T cells have been described but with contradictory results (31). It has been shown that Zol-treated moDCs triggered the expansion of γδ T cells with effector and costimulatory activities (32). Besides, moDCs pre-incubated with different ABP were able to stimulate the proliferation, activation, and secretion of IFNγ by γδ T cells (33) but with no impact on their cytotoxic activity (34, 35). Conversely, Vγ9Vδ2 T cell lines stimulated with HMB-PP strongly promote moDC maturation and IL12 secretion (33, 36). One study highlighted that TLR-L stimulated moDCs or pDCs trigger IFNγ secretion by Vγ9Vδ2 T cell subset (37), that in turn promote DC polarization into IL12p70-producing cells, and another one highlighted that polyI:C-stimulated CD11c+ DCs activate γδT cells via type I interferons (38), underlying the potential cross-talk between γδ T cells and PAMP-activated DCs. Most studies relied on the use of *ex-vivo* generated moDCs, and almost no data are available for pDCs.

pDCs and γδ T cells represent critical players in immunology to tumors and pathogens due to their unique properties and functional plasticity. Yet, interactions between these potent players have never been deeply studied. A better understanding of the interactions between pDCs and γδ T cells could allow their exploitation for immunotherapy. Here we investigated whether there is interplay between pDCs and γδ T cells, the nature of the response induced on pDCs or γδ T cells by the other partner, and the underlying molecular mechanisms. Co-culture of purified human pDCs and γδ T cells were performed in presence of TLR-L, PAg, and Zol (that will induce PAg accumulation) to mimic both tumor and infectious settings. Our study highlights crucial bidirectional pDC-γδ T cell interplay. Such understanding may help harnessing and synergize the power of pDCs and γδ T cells to fight against cancer and infections. These findings will pave the way to manipulate these potent and promising cell partners to design novel immunotherapeutic strategies.

## Materials and Methods

### Healthy Donor (HD)' Samples

Blood samples were obtained from 286 healthy volunteers. PBMCs were purified by Ficoll-Hypaque density-gradient centrifugation (Eurobio) and stored frozen in liquid nitrogen until use. All procedures were approved by the Ethics committee of the French Blood Agency's Institutional Review Board and declared under the reference #DC-2008-787. All participants gave written informed consent in accordance with the Declaration of Helsinki.

### Purification of pDCs and γδ T Cells

pDCs and γδ T cells were purified using, respectively, EasySep Human pDC enrichment kit and EasySep Human γδ T-cell enrichment kit (StemCell) according to manufacturer' instructions. The purity obtained was routinely above 90.5% for pDCs and 95% for γδ T cells.

### Tumor Cell Lines

Human melanoma lines COLO829 and A375 were purchased from ATCC (LGC-Standards). Cultures were performed in RPMI1640-Glutamax (Invitrogen) supplemented with 1% non- essential amino-acids, 1 mM sodium pyruvate (Sigma), 100 μg/ml gentamycin and 10% fetal calf serum (FCS) (Invitrogen).

### pDCs- γδ T Cells Coculture Experiments

Purified pDCs and γδ T cells were resuspended at 2 × 10^6^/ml in complete RPMI 1640 10% FCS and cocultured in a 1:1 ratio 20 h at 37°C, 5% CO_2_ (1 × 10^6^/ml final for each cell subset). Cocultures were performed as indicated in absence or presence of TLR7L (CL097, 1 μg/mL), TLR9L (CpG_A_, 1.5 μM) (Invivogen) and/or zoledronate (38.1 μM) (Novartis) to activate pDCs, IPP (80 μM) or HMB-PP (200 nM) (Sigma) together with IL2 (0.1 UI/ml) (Peprotech) and/or zoledronate (38.1 μM) to activate γδ T cells. Controls with only one partner (pDCs or γδ T cells alone) were performed in the same conditions. In some experiments, pDCs and γδ T cells were physically separated in different chambers by performing cocultures in the HTS Transwell-96 plates displaying a 0.4 μm polycarbonate membrane (Corning). To assess the impact of pDCs on γδ T cells, pDCs together with the activators were put in the upper compartment and γδ T cells in the lower chamber. To analyze the effect of γδ T cells on pDCs, γδ T cells together with the activators were put in the upper compartment and pDCs in the lower chamber. In some experiments, pDCs or γδ T cells were first pre-incubated 20 min with the following blocking antibodies [functional grade quality, no azide/low endotoxin (NA/LE)] alone or in different mixtures before adding the other cell partner: anti-IFNAR2 (pbl assay), -TNFR1, -GITR, -IFNGR1 (Thermofischer), -OX40L, -PD1, -TNFR2 (R&D Systems), -LAG3 (Adipogen), -ICOSL (Invitrogen), -TIM3, -NKG2D, -NKp30 (Biolegend) (all at 10 μg/mL), anti-41BB (antibodies online) (at 1 μg/mL), or mouse IgG and/or Goat IgG control isotypes (10–40 μg/mL depending on the corresponding amount of specific antibodies mixed together) (Thermofisher). When indicated, pDCs or γδ T cells were first pre-incubated 2 h with anti-BTN3A blocking antibody (clone 103.2, Creative Biolabs) at 10 μg/mL or mouse IgG control isotype (10 μg/mL).

### pDC Restimulation

When assessing the impact of γδ T cells on pDCs, the ability of pDCs to respond to a subsequent TLRL stimulation was assessed following the first coculture. In this case, the γδ T cells-pDCs cocultures were harvested after 20 h, washed, counted and pDCs were resuspended at 1 × 10^6^/ml and further cultured 24 h in absence or presence of TLR7L (CL097, 1 μg/mL) or TLR9L (CpG_A_, 1.5 μM) (Invivogen).

### Phenotypic Analysis

The phenotype of pDCs and γδ T cells was assessed either in the basal state or upon the 20 h cocultures and after the subsequent restimulation for pDCs as indicated. Cell suspensions were labeled with anti-human antibodies and their isotype-matched controls in PBS 2% FCS. pDCs were defined as CD45+ HLA-DR+ BDCA4+. γδ T cells were identified as CD45+ CD3+ panTCRγδ+, and further divided into δ2+ and δ2- subsets. The activation status of the cells was determined using anti-CD40, -CD80 (Beckman), -CD86 (BD) Abs for pDCs, and anti-CD69 (BD), -CD25 (eBiosciences) antibodies for γδ T cells. The expression profile of immune checkpoints was analyzed using anti-OX40, -OX40L, -ICOS,−41BB,−41BBL, -PD1, -PDL1, -PDL2 (BD), -ICOSL, -TIM3, -CTLA4, -LAG3 (eBiosciences) Abs; activating and inhibitory NKR were depicted using NKG2D (BD), NKp30, NKp44 (Beckman) Abs. TRAIL expression was evaluated on pDCs using anti-TRAIL Abs (BD). The expression of BTN3A was assessed on pDCs and γδ T cells at the basal state and upon specific stimulation using anti-BTN3A Abs (clone BT3.1, Miltenyi Biotec). Anti-TNFαRI, -TNFαRII Abs (R&D Systems), and anti-IFNαRI, -IFNαRII Abs (Miltenyi Biotec) were used to assess the expression of the corresponding molecules on pDCs and γδ T cells at the basal state and upon specific stimulation. We analyzed either the percentage of positive cells or the mean fluorescence intensity (MFI) of the positive cells as indicated. Suspensions were analyzed by flow cytometry using a FACS CantoII and DIVA software (BD). To ensure quality control during the study, we performed a systematic standardization of the fluorescence intensities using cytometer setup and tracking beads (CST) (BD).

### Cytotoxic Activity

γδ T cells cytotoxic activity was evaluated by a CD107 degranulation assay and perforin measurement upon coculture with target cells. Upon pDCs-γδ T cells 20 h cocultures, the cells were washed, γδ T cells were counted and further co-cultured with melanoma tumor cells (COLO829, A375) in a 20:1 ratio for 5 h. Anti-human CD107a/b Abs (BD) were added at the start of the coculture together with GolgiSTOP (BD) for the last 4 h. The cells were then labeled with CD45, CD3, panTCRγδ, TCRδ2 Abs (BD) before flow cytometry analysis. Perforin secretion was evaluated in the coculture supernatants using Human Perforin (PRF1) ELISA kit (AbCam).

### Soluble Factors Dosage

Human soluble IL4, IL10, IL17-A, IFNγ, TNFα, IFNα, IP10, TGFβ, and granzyme B production were measured in the coculture supernatants by a Cytometric Bead Array assay (CBA, BD).

### Statistical Analysis

The statistical analyses were performed by Graph Pad Prism software using the Wilcoxon matched *t*-test combined with Bonferroni correction or Mann-Whitney unpaired *t*-test.

## Results

### TLR-L or Zol-Activated pDCs Trigger Activation and Functionality of γδ T Cells

**Figure 1:**
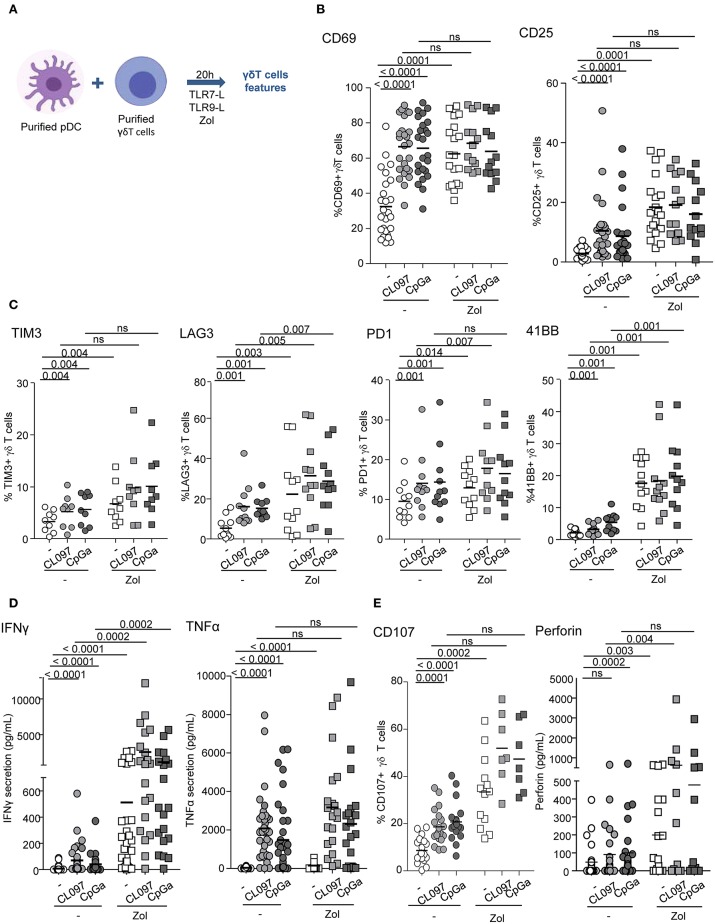
pDCs trigger phenotypic modulation and functional activity of γδ T cells. Purified pDCs and γδ T cells from healthy donors' blood were cocultured in absence (white dots) or presence of TLR7L (CL097) (light gray symbols), TLR9L (CpG_A_) (dark gray symbols) together with zoledronate (Zol) (square symbols) or not (round symbols). The phenotypic features of γδ T cells were depicted by flow cytometry. **(A)** Experimental scheme. **(B)** Activation status of γδ T cells evaluated by assessing CD69 and CD25 expression (*n* = 13–26). **(C)** Expression of selected immune checkpoints by γδ T cells: TIM3, LAG3, PD1, and 41BB (*n* = 7–11). **(D)** IFNγ and TNFα cytokine secretions were quantified in the culture supernatants by CBA (*n* = 20–30). **(E)** The cytotoxic activity of γδ T cells was evaluated through CD107 surface expression and perforin secretion upon subsequent coculture with melanoma tumor cells (*n* = 7–21). *P*-values were calculated using the Wilcoxon matched pairs test with Bonferroni correction.

We first evaluated the ability of pDCs to modulate the phenotype and functional activities of γδ T cells. Purified pDCs and γδ T cells from healthy donors' blood were co-cultured in absence or presence of TLR7L (CL097) or TLR9L (CpG_A_), together with Zol or not to promote accumulation of PAg and assess potential synergistic effect between TLR-L and Zol. Whole γδ T cells were used to investigate the ability of pDCs to impact on both δ2+ and δ2- subsets. The phenotypic and functional features of γδ T cells were then depicted (Figure 1A). When analyzing the whole population of γδ T cells, heterogeneity could rely on the fact that the frequencies of Vδ2+ and Vδ2- cells can differ considerably between donors, therefore we precisely analyzed each subset independently. pDCs drove a potent activation of γδ T cells as revealed by the upregulation of CD69 and CD25 activation markers following TLR7 or TLR9 stimulation, but also in presence of Zol (Figure 1B, Supplementary Figures 1A,B). These settings elicited also changes in immune checkpoint by γδ T cells as demonstrated by the upregulation of TIM3 (CD366), LAG3 (CD223), PD-1 (CD279), and 4-1BB (CD137) (Figure 1C, Supplementary Figure 1C). Other markers investigated [GITR (CD357), OX40 (CD134), ICOS (CD278), NKp30 (CD337), NKp44 (CD336), NKG2D (CD314)] were not significantly affected (Supplementary Figure 2A). In addition, TLR-L and/or Zol-treated pDCs elicited production of IFNγ and TNFα in cocultures (Figure 1D) and stimulated the cytotoxic activity of γδ T cells as illustrated by the upregulation of CD107 surface expression and secretion of perforin following culture with tumor cells (Figure 1E). These phenotypic and functional modulations were strongly induced on δ2+ T cells, whereas slightly triggered on δ2- T cells (Supplementary Figures 2B–E). We demonstrated that TLR7-/9-L drove a potent activation of pDCs associated with TRAIL expression and IFNα secretion, whereas Zol didn't have such impact on pDCs (Supplementary Figure 3A). The levels of modulation of γδ T cells induced by pDCs were similar to the one triggered by HMB-PP on γδ T cells alone, a strong stimulator of γδ T cells (Supplementary Figure 4A). Furthermore, we checked that, as expected, TLR7/9-L, Zol alone or in combination didn't have any direct impact on γδ T cells (Supplementary Figure 4B). Importantly, activation of pDCs was required as no modulation of γδ T cells was induced by unstimulated pDCs compared to γδ T cells cultured alone without pDCs (Supplementary Figure 5A). Altogether these data enlightened for the first time that TLR-L or Zol-activated pDCs trigger activation, cytokine secretion and cytotoxicity of γδ T cells.

### PAg-Activated γδ T Cells Drive Activation and Functionality of pDCs

**Figure 2:**
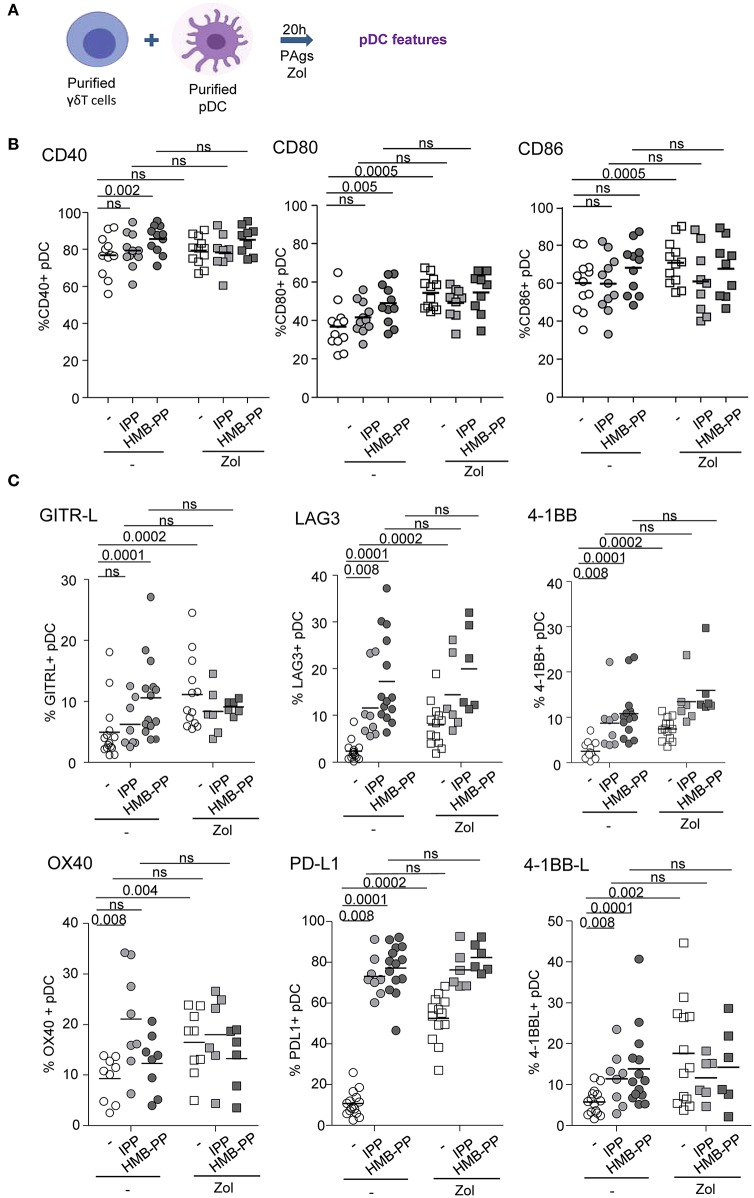
γδ T cells induce phenotypic modulation of pDCs. Purified γδ T cells and pDCs from healthy donor' blood were cocultured in absence (white dots) or presence of the phosphoantigens IPP (light gray symbols) or HMBPP (dark gray symbols) together with zoledronate (Zol) (square symbols) or not (round symbols). The phenotypic features of pDCs were depicted by flow cytometry. **(A)** Experimental scheme. **(B)** Activation status of pDCs evaluated by assessing CD40, CD80, and CD86 expression (*n* = 9–12). **(C)** Expression of selected immune checkpoints by pDCs: GITR-L, PDL1, LAG3, TIM3, 41BB, 41BB-L, and OX40 (*n* = 6–12). *P*-values were calculated using the Wilcoxon matched pairs test with Bonferroni correction.

**Figure 3:**
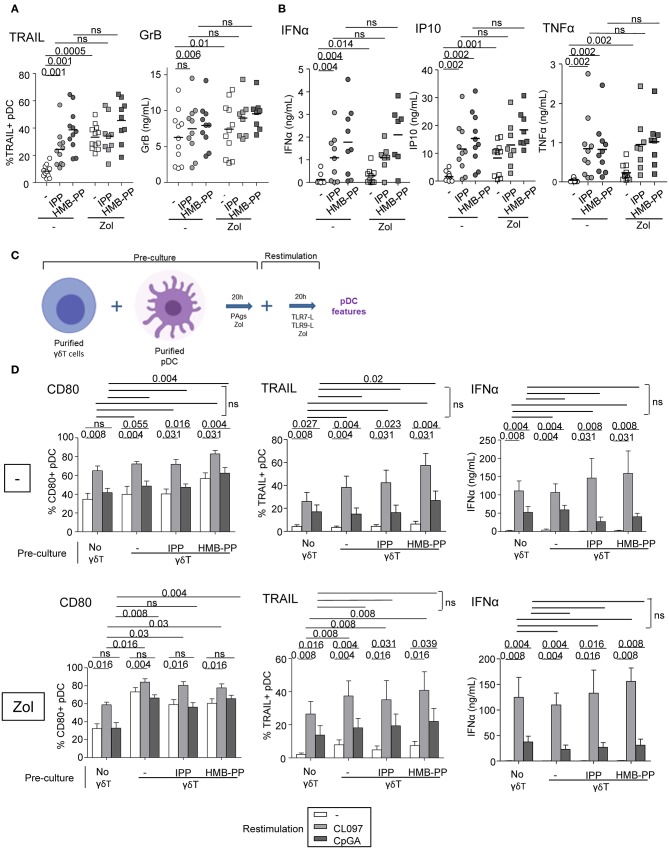
γδ T cells trigger functional activity of pDCs. Purified γδ T cells and pDCs from healthy donor' blood were cocultured in absence (white dots) or presence of the phosphoantigens IPP (light gray symbols) or HMBPP (dark gray symbols) together with zoledronate (Zol) (square symbols) or not (round symbols). **(A)** The cytotoxic capacity of pDCs was measured though expression of TRAIL and secretion of Granzyme B (*n* = 8–12). **(B)** IFNα, IP10, and TNFα cytokine secretions were quantified by CBA in the culture supernatants (*n* = 8–11). *P*-values were calculated using the Wilcoxon matched pairs test with Bonferroni correction. **(C,D)** The ability of pDCs to respond to a subsequent TLRL stimulation was evaluated by measuring CD80 expression, TRAIL exposure and IFNα secretion upon further culture in presence or not of TLR7L (CL097) or TLR9L (CpG_A_) and in absence (upper panels) or presence of Zol (lower panels) (*n* = 9). Bars represents mean ± SEM. *P*-values were calculated using the Wilcoxon matched pairs test.

We further examined the capacity of γδ T cells to modulate the phenotype and functional properties of pDCs. Purified γδ T cells and pDCs from healthy donors' blood were co-cultured in absence or presence of the phosphoantigens IPP or HMBPP together with Zol or not to promote accumulation of PAg and assess potential synergistic effect between PAgs and Zol. The phenotypic and functional features of pDCs were then depicted (Figure 2A). As the frequencies of Vδ2+ and Vδ2- cells can differ considerably between donors, this could bring some heterogeneity in the pDCs' features especially when Vδ2+-specific regimens are used. In the presence of HMB-PP or Zol but not IPP, γδ T cells induced the activation of pDCs as illustrated by the upregulation of CD40, CD80, and CD86 molecules (Figure 2B, Supplementary Figures 1D,E). PAg and Zol allowed γδ T cells to modulate the expression of some immune checkpoints on pDCs, especially GITR-L, 41BB/41BB-L, PDL1, LAG3, and OX40 (Figure 2C, Supplementary Figure 1F). Notably, PAg and/or Zol-treated γδ T cells elicited TRAIL upregulation on pDCs and production of IFNα and IP-10 (CXCL10) compared to γδ T cells—pDCs cocultured in absence of stimulation (Figures 3A,B). Granzyme B and TNFα could be produced both by pDCs or γδ T cells, but their levels were improved in presence of PAg and/or Zol. Furthermore, following coculture with PAg- and/or Zol-activated γδ T cells, the ability of pDCs to respond to a subsequent TLR7/9L stimulation was enhanced, as attested by increased CD80 expression and TRAIL exposure compared to coculture with unstimulated γδ T cells or to pDCs which didn't previously contact γδ T cells (Figures 3C,D). Such boosting of pDCs occurred in absence of Zol, only for HMB-PP-activated γδ T cells and TLR9-triggering of pDCs, and in presence of Zol, for unstimulated or activated γδ T cells and restimulation of pDCs with both TLR7-L and TLR9-L. However, it is worth mentioning that the ability of pDCs to produce IFNα was not improved by previous co-culture with γδ T cells. We demonstrated that HMB-PP drove a potent activation of γδ T cells associated with IFNγ secretion and CD107 exposure (Supplementary Figure 4A), whereas Zol didn't have such impact on γδ T cells (Supplementary Figures 4B,C), even so both activators were able to drive γδ T cell-dependent pDCs activation (Figure 2B). The levels of modulation of pDCs induced by γδ T cells were lower compared to the one triggered by TLR7/9L, strong stimulators of pDCs (Supplementary Figure 3A). Furthermore, we checked that HMB-PP, Zol alone or in combination didn't not have any direct impact on pDCs (Supplementary Figure 3B). Importantly, activation of γδ T cells was required as no evident modulation of pDCs was induced by unstimulated γδ T cells compared to pDCs cultured alone without γδ T cells (Supplementary Figure 5B). During γδ T cell/pDC cocultures, IFNγ was produced only in presence of phosphoantigens but not Zol alone (Supplementary Figure 5C). Thus, these observations unprecedently suggest that PAg-activated γδ T cells elicit pDCs' activation and functionality, even enhancing their potentialities.

### Both Soluble Factors and Membrane Contacts Are Required for pDCs—γδ T Cells Reciprocal Interplays

**Figure 4:**
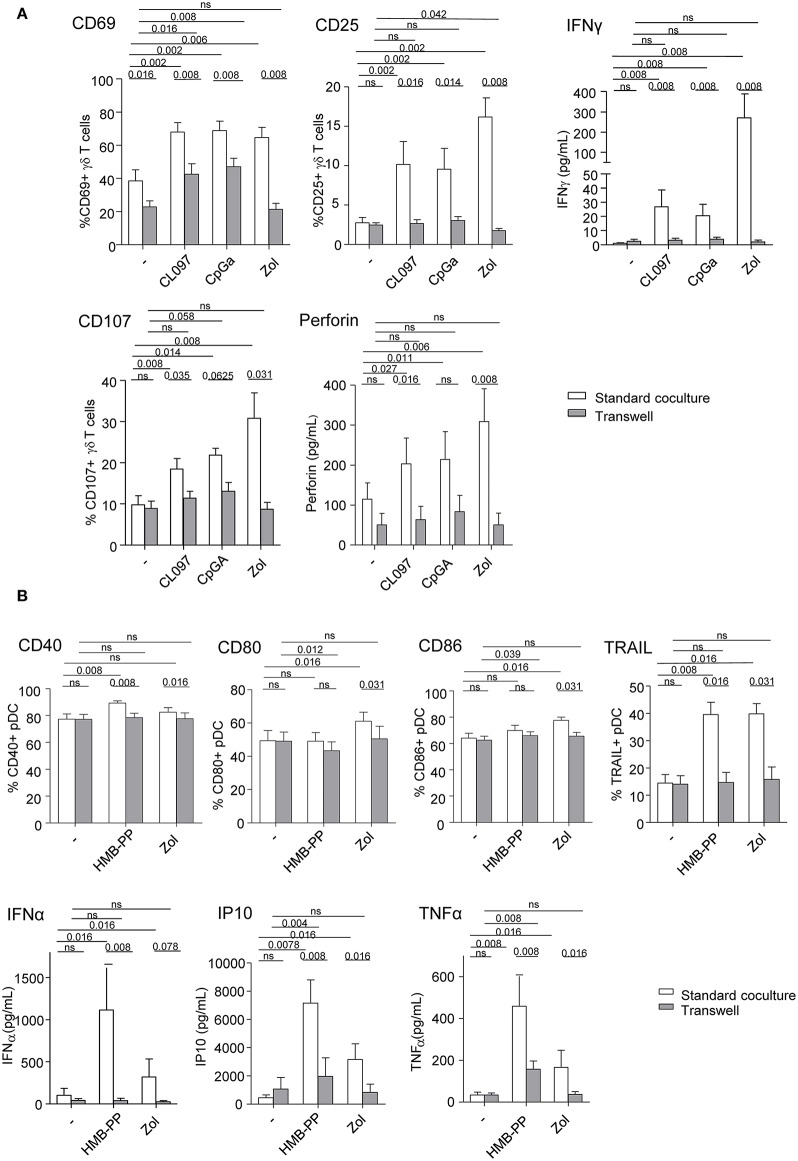
Membrane contacts and soluble factors are required for effective pDCs and γδ T cells cross-talk. Purified pDCs and γδ T cells from healthy donor' blood were cocultured together in the same well (white bars) or physically separated by a 0.4 μm membrane (transwell, gray bars) in absence or presence of TLR7L (CL097), TLR9L (CpG_A_), HMBPP or zoledronate (Zol) as indicated. The phenotypic and functional features of γδ T cells **(A)** and pDCs **(B)** were compared to identify the requirement for membrane contacts and/or soluble factors for their cross-talks. **(A)** CD69 and CD25 expression as well as IFNγ secretion by γδ T cells (upper panels, *n* = 8); CD107 surface exposure and perforin secretion by γδ T cells upon subsequent coculture with melanoma tumor cells (lower panels, *n* = 8). **(B)** Activation status of pDCs evaluated by measuring CD40, CD80, and CD86 expression (*n* = 8) and TRAIL expression by pDCs (upper panels, *n* =8); IFNα, IP-10, and TNFα cytokine secretion in the supernatants of cocultures (lower panels, *n* = 8). Bars represents mean ± SEM. *P*-values were calculated using the Wilcoxon matched pairs test.

We next investigated the implication of soluble factors and cell-cell contacts in the bidirectional cross-talk between pDCs and γδ T cells. Co-cultures were performed by physically separating the cells using Transwells (0.4 μm pores). In transwell conditions, the activation of γδ T cells triggered by TLR7/9-stimulated pDCs was partially (CD69) or totally (CD25) abrogated while IFNγ secretion and cytotoxicity were almost totally inhibited (Figure 4A). Similar results were obtained for δ2+ and δ2- T-cell subsets (Supplementary Figure 6). This suggests that both soluble mediators and membrane contacts are required for pDCs to tune γδ T cells. Furthermore, the modulation of pDCs elicited by PAg-activated γδ T cells didn't occur in transwell conditions, as revealed by the absence of upregulation of activation molecules, TRAIL exposure and IFNα secretion, even though IP-10 and TNFα secretions were strongly reduced but not totally (Figure 4B). These observations demonstrated that γδ T cells use mostly cell contacts to modulate pDCs, and to a lesser extend soluble mediators. In presence of Zol, the reciprocal impact of pDCs and γδ T cells on each other was totally abrogated when cells were physically separated (Figures 4A,B), suggesting exclusive cell-cell contacts. These results indicate that pDCs and γδ T cells cross-regulate each other through soluble factors and membrane contacts, the TLRL pathway requiring both signals whereas the PAg/Zol pathway mostly required cell contacts.

### pDCs and γδ T Cells Express BTN3A

**Figure 5:**
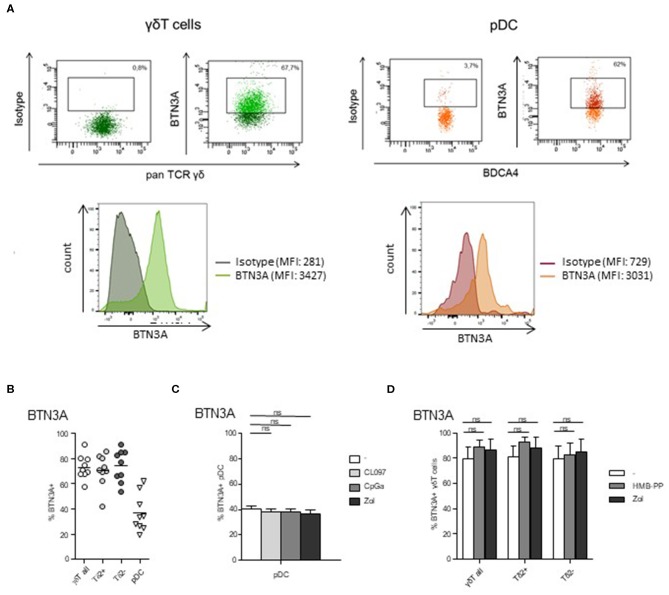
BTN3A is expressed by pDCs and γδ T cells at basal state and remained stable upon specific stimulation. The expression of BTN3A was evaluated by flow cytometry on pDCs and γδ T cells within PBMC of healthy donor at basal state **(A,B)** and on purified pDCs and γδ T cells upon specific stimulation **(C,D)**. **(A)** Representative flow cytometry analysis of BTN3A expression by γδ T cells and pDCs. Upper panels: dot plots gated on γδ T cells or pDCs; lower panels: histograms of BTN3A expression or control isotype on γδ T cells or pDCs. **(B)** Basal expression of BTN3A by γδ T cells (including δ2+ and δ2- T-cell subsets) and pDCs (*n* = 9). **(C)** Expression of BTN3A by purified pDCs either unstimulated or activated by TLR7L (CL097), TLR9L (CpGA), or zoledronate (Zol) (*n* = 6). **(D)** Expression of BTN3A by purified γδ T cells either unstimulated or activated by HMB-PP or zoledronate (Zol), both δ2+ and δ2- T-cell subsets were analyzed (*n* = 6). Bars represents mean ± SEM. *P*-values were calculated using the Wilcoxon matched pairs test with Bonferroni correction.

The BTN3A molecule is known to mediate the activation of γδ T cells by PAg. As its expression by pDCs is totally unknown, we next examined the expression of BTN3A by pDCs as well as by γδ T cells. We found that BTN3A was expressed by around 40% of pDCs (Figures 5A,B), and this level was not modulated by TLR7/9-L or Zol (Figure 5C). As expected, γδ T cells, including both δ2+ and δ2- T-cell subsets, also express BTN3A for 80% (Figures 5A,B) whose level stays stable in presence of HMB-PP or Zol (Figure 5D). Thus, Zol-treated pDCs, by accumulating PAg, are well-equipped to activate γδ T cells through BTN3A/PAg complexes.

### TLR-L-Dependent pDC-Induced γδ T Cells Activation Requires Soluble IFNα, TNFα, Membrane OX40L and Slightly BTN3A, Whereas Zol-Dependent pDC-Induced γδ T Cells Full Potency Necessitates Mostly BTN3A and IFNα

**Figure 6:**
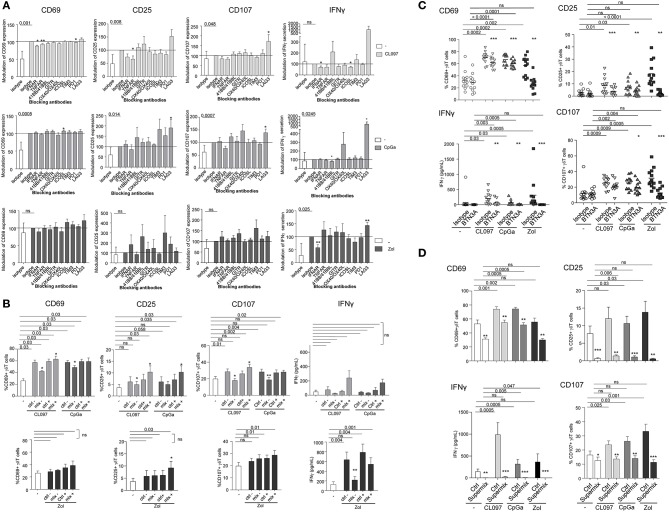
Revealing the molecular mechanisms involved in the pDCs- γδ T cells cross talk. Purified pDCs from healthy donors' blood were pre-incubated with single or mixtures of blocking antibodies and cocultured with purified γδT cells in the presence or not of TLR7-L (CL097) (light gray bars/symbols), TLR9-L (CpG_A_) (dark gray bars/symbols) or zoledronate (Zol) (black bars/symbols) as described in Figure S7. The features of γδT cells were then depicted: the activation status (CD25 and CD69 expression) and the cytotoxic activity (CD107 surface exposure) were analyzed by flow cytometry; IFNγ secretion was measured by CBA in the supernatants. **(A)** Blocking with single antibodies: anti-IFNAR2, -TNFAR1/TNFAR2, -ICOS-L, -TIM3, -PD1,−41BB/41BB-L, -GITR, -LAG3, OX40/OX40-L antibodies. Results were standardized toward the stimulated isotype-matched control condition (*n* = 12–16). **(B)** Blocking with a mixture of antibodies. For TLR7/9L and Zol stimulation, the “mix –” is composed of anti-IFNAR2, -TNFR1/TNFR2, and -OX40 antibodies, and the “mix +” composed of anti-TIM3, -PD-1,−4-1BB, -GITR antibodies (*n* = 6–10). The groups “ctrl –” and “ctrl +” correspond to the mixture of isotype control Abs corresponding, respectively, to the “mix –” and “mix +” groups. **(C)** Blocking with single anti-BTN3A antibodies (*n* = 12). **(D)** Blocking with a Supermix composed of the inhibitory mixture (mix—as in **B**) together with anti-BTN3A blocking antibody (*n* = 12). Bars represents mean ± SEM. *P*-values were calculated using the Wilcoxon-matched pairs test. Lines: comparison between the stimulated condition and the unstimulated one. Stars: comparison within the stimulated conditions between the specific blocking and the corresponding control isotype (^*^p < 0.05, ^**^p < 0.01, ^***^p < 0.001); only significant statistics are reported.

To further gain insight into the mechanism of pDCs—γδ T cells interplay, we explored the nature of the soluble factors and membrane molecules required for pDCs to modulate γδ T cells. For each condition of stimulation (TLR7L, TLR9L, Zol), we performed the co-culture in presence of antibodies blocking the receptors for soluble factors or by pre-incubating pDCs with blocking antibodies directed toward specific surface molecules (Supplementary Figure 7A). Molecules were selected based on their expression by pDCs and/or modulation of expression upon TLR7/9 triggering (data not shown), and on the expression of the corresponding receptors on γδ T cells. Hence, IFNα and TNFα could be potential soluble candidates, and ICOSL, TIM3, PDL1, 41BB, GITRL, LAG3, OX40L, and BTN3A possible membrane candidates. γδ T cells express some levels of ICOS, PD1, 4-1BB, GITR, and OX40 (Figure 1C, Supplementary Figure 2A). We evaluated the expression of TNFαRI, TNFαRII, and IFNαRI/RII on both partners. Under steady state, pDCs expressed TNFαRI, TNFαRII and high level of IFNαRII, whereas γδ T cells expressed low levels of TNFαRI, TNFαRII, and IFNαRII (Supplementary Figure 8A). During pDC/ γδ T-cell co-cultures (Supplementary Figure 8B), we observed a down-modulation of TNFαRII and IFNαRII on pDCs in presence of HMB-PP, CLO97, or CpGA probably due to the presence of the corresponding cytokine on the receptors and/or its modulation of expression by the cytokine. The expression of TNFαRII on γδ T cells increased in presence of HMB-PP, whereas the expression of IFNαRII remained low. These results suggest that TNFα and IFNα could potentially trigger signaling in γδ T cells. We observed that single blocking of IFNAR, TNFAR, or OX40L inhibited at least one of the pDC-induced features of γδ T cells following TLR7-L stimulation or Zol addition but not TLR9 triggering, whereas single blocking of ICOSL, TIM3, PDL1, 41BB, GITRL, or LAG3 had rather an enhancing impact (Figure 6A). Interestingly, the blocking of some immune checkpoint, especially LAG3, dramatically enhanced the activation and functionality of γδ T cells induced by pDCs. By mixing the blocking antibodies displaying a negative (“mix –”) or positive (“mix +”) impact, the effect on γδ T cells' potentialities was stronger: the activation, IFNγ secretion and/or cytotoxicity of γδ T cells in presence of TLR7/9L were significantly inhibited by simultaneously blocking IFNα, TNFα, and OX40L, whereas γδ T cells' features were enhanced in presence of the mixture of anti-ICOSL, TIM3, PDL1, 41BB, GITRL, and LAG3 antibodies (Figure 6B). In Zol condition, only IFNγ secretion was strongly abrogated by the “mix –.” Very interestingly, the features of γδ T cells induced by TLR7/9L-activated pDCs were slightly but significantly impacted by the blocking of BTN3A, whereas this setting totally abrogated Zol-dependent triggering of γδ T cells (Figure 6C). We then elaborated a “Supermix” by combining the mixture of antibodies displaying an inhibitory impact (IFNAR, TNFAR and OX40L) together with anti-BTN3A1 antibodies. Strikingly, the Supermix totally abrogated the activation, IFNγ secretion and cytotoxicity of γδ T cells triggered by TLR7/9L- or Zol-activated pDCs (Figure 6D). Importantly, blocking of BTN3A alone or use of the Supermix didn't impact the ability of pDCs to upregulate activation molecules or secrete IFNα in response to TLR7/9L stimulation (Supplementary Figure 9). By directly comparing the BTN3A and Supermix conditions (Supplementary Figure 10), we observed that, in presence of TLR-L, BTN3A has a slight inhibitory impact that can be further improved by the blocking of the molecules targeted by the Supermix, whereas in presence of Zol, BTN3A is the major player involved in the cross-talk as its effect cannot be further improved by the Supermix. Thus, these observations revealed that TLR-L-dependent pDC-induced γδ T cells triggering required soluble IFNα, TNFα, membrane OX40L and slightly BTN3A, whereas Zol-dependent pDC-induced γδ T cells full potency relies mostly on BTN3A and IFNα.

### PAg-Dependent γδT Cells-Induced pDC Stimulation Requires Exclusively BTN3A and IFNγ

**Figure 7:**
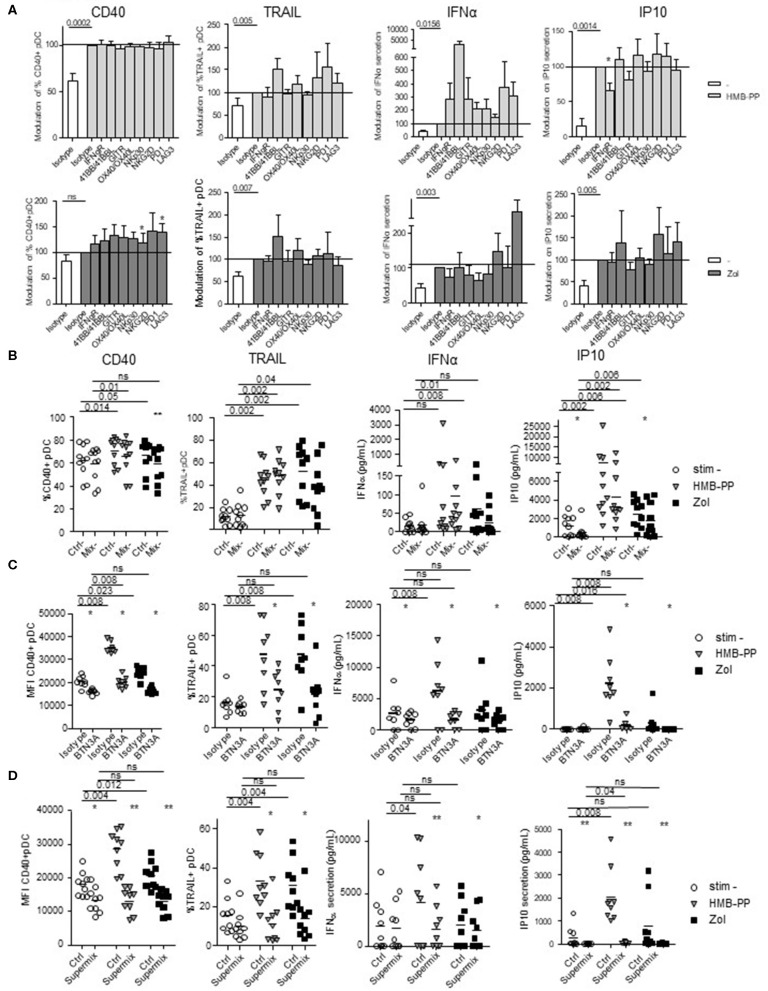
Revealing the molecular mechanisms involved in the γδ T cells-pDCs cross talk. Purified γδT cells from healthy donor' blood were pre-incubated with single or mixtures of blocking antibodies and cocultured with purified pDCs in the presence or not of phosphoantigen (HMBPP) (light gray bars/symbols) or zoledronate (dark gray bars/symbols). The features of pDCs were then depicted: the activation status (CD40) and cytotoxic properties (TRAIL) were analyzed by flow cytometry, whereas the cytokine secretion (IFNα, IP10) was assessed by CBA in the supernatants. **(A)** Blocking with single antibodies: anti-IFNγR,-NKG2D,-NKp30,-PD-1,-41BB/41BB-L,-GITR,-LAG3,-OX40/OX40-L antibodies. Modulations were standardized toward the isotype-matched control condition (*n* = 15). Bars represents mean ± SEM. **(B)** Blocking with a mixture of blocking antibodies composed of anti-IFNγR, -NKp30, and -GITR antibodies (*n* = 10). **(C)** Pre-activated γδT cells with HMB-PP and Zol during 4 h were mixed with pDCs pre-blocked with single anti-BTN3A for 2 h (*n* = 8). **(D)** Cultures were performed in presence of the Supermix composed of the inhibitory mix together with anti-BTN3A. γδT cells were pre-activated with HMB-PP and Zol during 4 h in presence of the inhibitory mix for the last 2 h and mixed with pDCs pre-blocked with single anti-BTN3A for 2 h (*n* = 9). Lines: comparison between the stimulated condition and the unstimulated one. Stars: comparison within the stimulated conditions between the specific blocking and the corresponding control isotype (^*^p < 0.05, ^**^p < 0.01; only significant statistics are reported.

To decipher the mechanism of γδ T cells—pDCs interplay, we further investigated the nature of the soluble factors and membrane molecules required for γδ T cells to modulate pDCs. For each condition of stimulation (HMB-PP, Zol), we performed the co-culture in presence of antibodies blocking the receptors for soluble factors or by pre-incubating γδ T cells with blocking antibodies directed toward specific surface molecules (Supplementary Figure 7B). Molecules were selected based on their expression by γδ T cells and/or modulation of expression upon HMB-PP stimulation (data not shown). Hence, IFNγ could be a potential soluble candidate, and NKG2D, NKp30, PD1, 41BB, GITR, LAG3, OX40, and BTN3A1 possible membrane candidates. We observed that single blocking of IFNγR inhibited IP-10 secretion upon HMB-PP stimulation (Figure 7A). The single blocking of NKp30 and GITR had also a slight negative impact on at least one of the pDC-induced features of γδ T cells, whereas single blocking of 41BB, NKG2D, or LAG3 had rather an enhancing impact in both HMB-PP and Zol conditions (Figure 7A). By mixing the blocking antibodies displaying a negative (“mix –”) impact, the effect on pDCs' potentialities was stronger: the activation, TRAIL exposure, IFNα and IP10 secretion of pDCs in presence of HMB-PP or Zol were significantly inhibited by simultaneously blocking IFNγ, NKp30, and GITR (Figure 7B). However, by mixing the blocking antibodies displaying a positive (“mix +”) impact (anti-41BB, -NKG2D, and -LAG3 antibodies), pDCs' features were not modified in presence of the mixture (not shown). Importantly, the use of the mix—didn't impact the ability of γδ T cells to upregulate activation molecules, secrete IFNγ or exhibit cytotoxicity in response to HMB-PP or Zol stimulation (Supplementary Figure 11A). As the blocking of BTN3A1 directly on γδ T cells prevented their activation in response to HMB-PP (not shown), we pre-activated γδ T cells with HMB-PP and Zol during 4 h before mixing them with pDCs pre-blocked with anti-BTN3A Abs to assess the involvement of BTN3A in the γδ T cells-pDC cross-talk. In these settings, γδ T cells responded to HMB-PP stimulation even though to a lesser extent than in control conditions (Supplementary Figure 11B). Strikingly, in these settings, the features of pDCs induced by HMB-PP-activated γδ T cells were partially (for CD40 and IP10 secretion) or totally (for TRAIL and IFNα secretion) abrogated by the blocking of BTN3A1, whereas the inhibitory effect of BTN3A blocking was total for all parameters in the presence of Zol (Figure 7C). Cultures were then performed in presence of the Supermix composed of the inhibitory mix together with anti-BTN3A antibodies. γδ T cells were pre-activated with HMB-PP and Zol during 4 h in presence of the inhibitory mix for the last 2 h and mixed with pDCs pre-blocked with single anti-BTN3A antibodies for 2 h. Remarkably, pDCs activation, TRAIL expression, IFNα and IP10 secretion were totally abrogated in presence of the Supermix (Figure 7D). By directly comparing the BTN3A and Supermix conditions (Supplementary Figure 12), we observed that BTN3A has a major inhibitory impact that can be slightly further increased by the blocking of the molecules targeted by the Supermix (especially for CD40, TRAIL, and IP10). Altogether, these observations enlightened that HMB-PP/Zol-dependent γδ T cells—induced pDCs triggering required mostly soluble IFNγ and membrane BTN3A1. Such interplay could be further enhanced by NKp30 and GITR.

## Discussion

**Figure 8:**
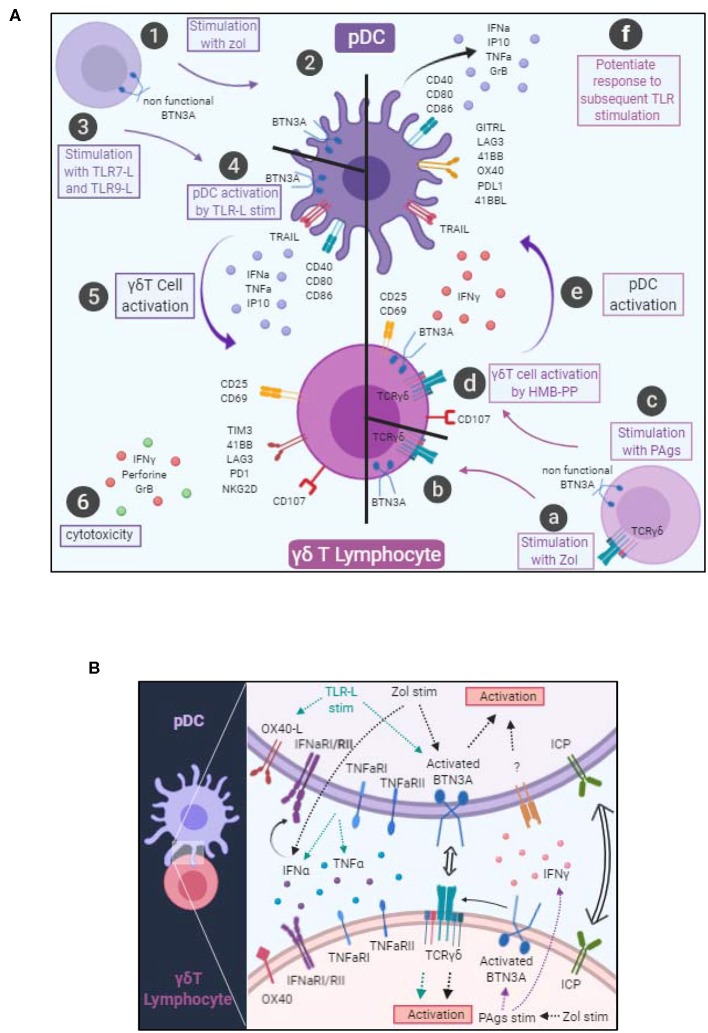
Graphical summary. **(A)** Cellular interactions between pDCs and γδ T cells. pDCs stimulated with Zol probably undergo a BTN3A conformational change (steps 1–2). pDCs stimulated with TLR7L or TLR9L upregulate CD40, CD80, CD86 and secrete IFNα, TNFα, and IP10 (steps 3–4). These changes drive γδ T cell activation, modulation of immune checkpoint expression together with IFNγ secretion and trigger their cytotoxic potential (steps 5–6). γδ T cells stimulated in presence of Zol (steps a–b) or with PAgs (step c) probably undergo a BTN3A conformational change that subsequently elicit activation molecules, cytotoxic potential and IFNγ secretion (step d). Such modifications trigger pDC activation, modulation of immune checkpoint expression together with secretion of IFNα, TNFα, and IP10 and upregulation of TRAIL (step e), and potentiate their response to a subsequent TLR-L triggering (step f). **(B)** Molecular mechanisms involved in the bidirectional cross-talk between pDCs and γδ T cells. pDC-triggered γδ T cell activation involves soluble factors (IFNα, TNFα) and surface molecules (OX40L and BTN3A). γδ T cell-triggered pDC activation involves soluble factors (IFNγ) and surface molecules (BTN3A). Both cross-talks can be modulated negatively or positively by immune checkpoints.

Based on their outstanding features, both pDCs and γδ T cells can be considered as sentinels and orchestrators of immune responses, able to trigger and orientate immune responses against pathogens and tumors. However, these two actors can be corrupted by pathogens or tumor cells, leading to pathogenic immune responses. We explored the interplay between pDCs and γδ T cells and highlight for the first time the existence of reciprocal interactions between these key potent immune players, deciphering the mechanisms underlying such cross-talk (Figure 8).

We demonstrate that TLR7/9L- or Zol-stimulated pDCs drive potent γδ T cells activation, enhancing Th1 cytokine secretion and cytotoxic activity. We also show that γδ T cells activated by PAg trigger pDC phenotypic changes and elicit their functional activities. Moreover, we provide evidence that these interactions require cell-cell contact and soluble factors, and identify the underlying mechanism of their interplay. The features of γδ T cells triggered by pDCs activated by ABP (Zol) or TLRL were comparable in intensity to the DC-independent ones elicited by PAg such as HMB-PP. However, while PAg activate only the Vδ2+ T cell subset, pDCs were able to additionally activate the Vδ2- T cell subset. Interestingly, we point out that γδ T cells activated by pDCs displayed some differences compared to γδ T cells activated by moDC. Indeed, previous studies, mostly relying on the use of *ex-vivo* generated moDCs, highlighted that in presence of Zol, moDCs can induce the activation, proliferation and immunoregulatory functions of γδ T cells without enhancing their cytotoxicity (34, 39). In turn, γδ T cells promote DC maturation and improve their capacity to trigger adaptive αβ T cell responses (3). We demonstrate that pDCs can elicit the cytotoxic function of γδ T cells, whereas most studies using moDCs revealed that cytotoxicity was not induced. Also, compared to previous studies that exclusively used ABP, we enlightened that TLRL-activated pDCs can drive potent γδ T cells' activation and functionality. This is in accordance with one study that demonstrated that mDCs and pDCs activated by TLR3L, TLR7/8L, or TLR9L can trigger IFNγ secretion by Vγ9δ2 T cells (37). In our hands, pDCs activate both Vδ2+ and Vδ2- T cells, provoke major phenotypic changes (upregulation of CD25 and CD69, modulation of immune checkpoint expression) and drive their cytotoxicity toward tumor cells. We also demonstrate that in turn IPP or HMB-PP stimulated-γδ T cells triggered major phenotypic and functional changes in pDCs, driving their activation (CD40, CD80, CD86), cytokine release (IFNα, IP10), and cytotoxicity (TRAIL upregulation). We used both IPP and HMB-PP to decipher whether phosphoantigens from different sources (bacterial/mammalian) could have similar effects on the pDC/ γδ T cell cross-talk. Even though IPP and HMB-PP exhibit the same potency on γδT/pDC cross-talk but with different magnitude, this is not a matter of doses because we chose the optimal dose for each of them, within the μM range for IPP and within the nM range for HMB-PP. HMB-PP is a more potent stimulator at doses 1,000 times inferior. Actually, it has been demonstrated that IPP and HMB-PP have a different affinity for the B30.2 intracellular domain of BTN3A, IPP exhibiting an affinity of 672 mM and HMB-PP was found to bind with an affinity of 1.1 mM, explaining the 1,000- to 10,000-fold difference in bioactivity of the two molecules (25, 40). Another study revealed that, upon a 6-day coculture, CpG_B_-activated pDCs triggered the proliferation of Vγ9δ2 T cells with a preferential expansion of the memory subsets (both central memory and effector memory), and induced their polarization toward IL-17 secretion (41). We also found a slight (but not significant) increase in IL17 secretion in cocultures of pDC- γδ T cells in presence of TLR7L+Zol compared to unstimulated cocultures (data not shown), which may confirm the tendency of pDCs to drive Th17-oriented γδ T cells. Notably, we also reported that pDC- γδ T cells interplay can be strongly enhanced or inhibited by blocking selective immune checkpoints, offering opportunities to further modulate and orientate the outcomes of these cross-talks.

We deciphered the mechanism of such interplay, and highlight differential TLRL- or Zol-dependent pathways. Previous reports have demonstrated that interplay between moDC and γδ T cells involved both soluble and membrane-bound signals. Such interaction was found to be dependent on PAg (IPP) (32, 42, 43), cytokines (IL12, IL15, TNFα, IFNα/β) (34, 37, 39) and cell contact (adhesion molecules CD54-CD11a, CD86-CD28) (33, 35). Reciprocally, Vγ9Vδ2 T cell lines stimulated with HMB-PP promote moDC maturation and IL12 secretion (33, 36), which was found to depend on CD1c and TNFα (44) or IFNγ (45) or cell contact (33). Neither immature moDCs nor their mature counterparts were capable of activating γδ T cells, but they acquired this ability when stimulated with zoledronate (32). We also point out that the cross-talk between pDCs and γδ T cells occurs only upon activation of one partner. Our study revealed that TLR-L dependent pDC-induced γδ T cells triggering required soluble IFNα, TNFα, membrane OX40L and slightly BTN3A, whereas Zol-dependent pDC-induced γδ T cells full potency necessitated mostly BTN3A and IFNα. ABP target the intracellular mevalonate pathway and by blocking the farnesyl pyrophosphate synthase induces the accumulation of metabolites (such as IPP). ABP triggers a DC-dependent activation of γδ T cells. Even though Zol had no direct effect on pDCs activation or cytokine secretion, it most likely elicits accumulation of IPP that further activate γδ T cells through BTN3A. It would be also critical to investigate the TCRγδ-dependency of activation of γδ T cells in pDC/γδ T cells cross-talk, even though we can expect, based on BTN3A blocking experiments, that the TCRγδ is likely to be slightly involved in the activation of the γδ T cells by TLR-L-stimulated pDCs, but strongly required in the pDC-dependent activation of γδ T cells in presence of Zol. We show for the first time that human pDCs express BTN3A molecules, hence being potent mediators of PAg-induced γδ T cells activation. Zol treatment of pDCs may lead to accumulation of IPP, which either provokes a conformational change of BTN3A or is released through ABCA1 transporter, mediating activation of γδ T cells. It has been recently demonstrated that intracellular IPP, accumulated following mevalonate pathway inhibition, is released by Zol-treated DCs through the ATP-binding cassette transporter A1 (ABCA1) in cooperation with apolipoprotein A-I (apo-I) and BTN3A1 (46). Regulation of BTN3A1 stability together with protein trafficking and expression of the transporter ABCA1 within tumor microenvironment, as shown for doxorubicin (47), may be crucial to trigger optimal cross-talks between DCs and γδ T cells. Conformational changes of BTN3A1 might represent a key step in the detection of infection or tumorigenesis by γδ T cells (48). Depending on the isoform, BTN3 molecules could have stimulatory or inhibitory activity, suggesting that they might be considered as novel immune checkpoint to be targeted to potentiate the anti-tumor/viral activity of γδ T cells (49). The mandatory role of BTN3A in pDCs/ γδ T cells bidirectional cross-talks that we highlight here renders it central in the regulation of immune responses toward infected or tumor cells, and very promising to design or optimize immunotherapeutic strategies.

Interestingly, it has been demonstrated that MICA and MICB can be expressed by DCs under certain conditions and function as activator ligands for NK and γδ T cells. Indeed, in response to IFNα, DCs are able to express MICA/B and activate NK cells following ligation of NKG2D (50), and Mycobacterium tuberculosis-infected DCs were shown to express MICA and subsequently activate Vδ2+ T cells (51). The ability of pDCs to express MICA/B still remains to be determined, but this is likely that pDCs may express MICA/B upon TLR-L stimulation that drive IFNα production, and further regulate γδ T cells through NKG2D.

Our study highlights that the interactions between pDCs and γδ T cells could play a role in the therapeutic activity of ABP. Such cross-talk may participate to the beneficial immune effects of Zol administration in cancer (9). ABP drugs used as adjuvant cancer therapy for the treatment of malignant osteolytic bone disease, could activate the anti-tumor effector functions of γδ T cells via pDCs. Interestingly, it has been shown in colorectal cancer that Zol can induce the expression of BTN3A1 within the tumor microenvironment thus stimulating effector γδ T cells with antitumor activity (52).

Therapies exploiting the potential of either pDCs or γδ T cells are currently emerging (53–55). Indeed, anti-cancer therapies can exploit the power of γδ T cells (56) through indirect stimulation by ABP (zoledronate, pamidronate) of Vδ2+ cells or adoptive transfer of *ex-vivo* expanded γδT cells that revealed promising clinical efficacy. Besides, pDCs can be mobilized by administration of TLRL leading to protective antitumor responses (57), or can be directly used as vectors for vaccination (11, 58, 59). The interplay between pDCs and γδ T cells could be exploited to achieve reciprocal activation and pave the way for novel immune-based strategies, influencing pDCs through modulation of γδ T cells and reciprocally affecting γδ T cells by impacting pDCs. Our findings provide rationale for combinatorial therapy engaging both cell types, by simultaneously targeting pDCs and γδ T cells using both TLR agonists and ABP for synergistic activity. The expanded knowledge on pDC—γδ T cells interactions brings opportunities for new immunotherapies harnessing their potential.

All studies including ours were performed *ex vivo*, but we can envision that pDCs and γδ T cells have the opportunity to meet and interact *in vivo* in pathophysiologic conditions. Indeed, pDCs can be located in blood and lymphoid tissues, and accumulate at inflammatory sites. γδ T cells, which are found in the circulation and in tissue (such as epidermis, dermis, intestine, lung, uterus), express chemokine receptor allowing them to migrate to inflamed tissues (CXCR3, CCR5) or lymph nodes (CCR7, CD62L) (60) and are able to migrate to sites of infections (61) where they could meet pDCs. Furthermore, both pDCs and γδ T cells infiltrate tumors and could meet within tumor microenvironment. Strikingly, there are *in vivo* evidences that the cross-talk between DCs and γδ T cells is involved in many physiopathological situations. For example, the DC/ γδ T cells cross-talk can be exploited by pathogens for immune evasion. HIV-infection of moDCs inhibits Vγ9Vδ2 T cell functions (proliferation, cytokine production) and reciprocal DC activation (62). On the opposite, DC/ γδ T cells cross-talk can initiate/boost immune responses to pathogens. γδ T cells promote the maturation of DCs and subsequent T-cell priming during West Nile Virus infection (63), and BCG-infected DCs can prime and expand cytotoxic γδ T cells (39). Such DC/ γδ T cells cross-talk is also central in host-microbiota interactions as shown for microbiota-activated CD103+ DCs that can elicit γδT17 (64) and therefore is central in IL17-driven inflammatory diseases. In addition, DCs activated by ABP can empower γδ T cells with anti-tumor immunity (43, 65, 66) (activation of γδ T cells in turn elicit TAA-specific CD8 T cell responses). The cross-talk between pDCs and γδ T cells has never been address *in vivo*. Investigating γδ T cell function in mice deficient for pDCs, and pDC function in mice deficient for γδ T cells in the context of infection or cancer would provide significant relevance for the phenomena that we described *in vitro*. The understanding of the cross-talk between DCs and γδ T cells is crucial to better exploit it for immunotherapy.

Together, pDCs and γδ T cells have strategic locations covering all epithelial barriers (skin, intestine, lung), circulation and lymph nodes, and sense all potential danger directly on pathogens or on infected/transformed cells through PRR or TCR by recognizing different types of PAMPs, thereby widening the scope of immune responsiveness. pDCs and γδ T cells display a large panel of cytokine secretion especially type I and II IFN, offer distinct combinations of functional potentials, and may finally decide the tolerogenic or immunogenic nature of the response to elaborate. The cross-talk between these key cellular partners may help refining the type of immune response to elicit to precisely adjust it to the danger to face. Knowing the crucial involvement of pDCs and γδ T cells in immunopathologies (cancer, infections, autoimmunity) and given their key role in bridging innate and adaptive immunity through regulation of any other cell function, a better understanding of the interplay between pDCs and γδ T cells can bring opportunities to control the outcome of immune responses against tumors, pathogens, and autoantigens. Our study reveals that pDC and γδ T cells have the capacity to harness each other potential and to synergise through diverse pathways involving soluble factors and membrane contacts. Acting on such synergy represents a promising way to restore appropriate immune responses in cancers, infections, and autoimmune diseases. These fascinating cell types with unique and crucial functions unveil an additional facet of their potential.

## Data Availability Statement

All datasets generated for this study are included in the article/Supplementary Material.

## Ethics Statement

The studies involving human participants were reviewed and approved by French Blood Agency's Institutional Review Board (reference #DC-2008-787). The patients/participants provided their written informed consent to participate in this study.

## Author Contributions

CA conceived the project and directed research. CA and PG designed the experiments and wrote the manuscript. PG, BP, and CA performed the experiments. PG, BP, CA, and LC analyzed the data. LC and JC provided research input and contributed to data interpretation and manuscript editing.

## Conflict of Interest

The authors declare that the research was conducted in the absence of any commercial or financial relationships that could be construed as a potential conflict of interest.
