# Supplementary material for: Potent Bidirectional Cross-Talk Between Plasmacytoid Dendritic Cells and γδT Cells Through BTN3A, Type I/II IFNs and Immune Checkpoints
Source: Front Immunol. 2020 May 6;11:861. doi: 10.3389/fimmu.2020.00861 (PMC7218166; doi:10.3389/fimmu.2020.00861)

Supplementary Material

1 Supplementary Figures

Supplementary Figure 1. Flow cytometry gating strategy

Purified pDCs and γδ T cells from healthy donors' blood were cocultured in absence or presence of TLR7L (CL097), TLR9L (CpG_A), phosphoantigens IPP or HMBPP together with zoledronate (Zol) or not. The phenotypic features of pDCs or γδ T cells were depicted by flow cytometry. (**A-C**) Upon pDC stimulation, γδ T cell features were defined: γδ T cells were defined using CD45, CD3, pan γδ TCR and δ2 markers (**A**), and CD69, CD25 and CD107 expression were further analyzed (**B**) together with immune checkpoints (LAG3 is shown) (**C**). (**D-F**) Upon γδ T cell activation, pDC features were depicted: pDCs were determined using CD45, HLA-DR and BDCA4 markers (**D**), and expression of CD40, CD86 and TRAIL was analyzed (**E**) together with immune checkpoints (PDL1 is shown) (**F**).

Supplementary Figure 2. pDCs trigger phenotypic modulation of γδ T-cell subsets

Purified pDCs and γδ T cells from healthy donors' blood were cocultured in absence (white dots) or presence of TLR7L (CL097) (light gray symbols), TLR9L (CpG_A) (dark gray symbols) together with zoledronate (Zol) (square symbols) or not (round symbols). The phenotypic features of γδ T cells including δ2+ and δ2- subsets were depicted by flow cytometry. (**A**) Expression of selected immune checkpoints and NKR by γδ T cells: GITR, OX40, NKp30, NKp44 and ICOS (n= 7 to 11). (**B-C**) Activation status of δ2+ and δ2- T-cell subsets evaluated by assessing CD69 (**B**) and CD25 (**C**) expression (n=13 to 26). (**D**) Expression of selected immune checkpoints and NKR by δ2+ and δ2- T-cell subsets: TIM3, LAG3, PD-1, 41BB, and NKG2D (n=7 to 11). (**E**) The cytotoxic activity of δ2+ and δ2- T-cell subsets was evaluated through CD107 surface expression upon subsequent coculture with melanoma tumor cells (n=7 to 21). P-values were calculated using the Wilcoxon matched pairs test with Bonferroni correction.

Supplementary Figure 3. TLR7/9L stimulation triggers pDC activation while Zol and HMB-PP have no direct impact on pDC' features

Purified pDCs from healthy donors' blood were cultured with or without (**A**) TLR7L (CL097), TLR9L (CpGA) or zoledronate (Zol) (n=9 to 11) or (**B**) HMB-PP alone or combined with Zol (n=4). The phenotypic and functional features of pDCs were then assessed through CD40, CD80 and CD86 expression, TRAIL exposure and IFNα secretion. Bars represents mean±SEM. P-values were calculated using the Wilcoxon matched pairs test with Bonferroni correction.

Supplementary Figure 4. HMB-PP elicits activation and functionality of γδ T cells while TLR7/9L and Zol have no direct impact on γδ T cells' features

Purified γδ T cells from healthy donor' blood were cultured with or without (**A**) HMB-PP (n=13 to 25) or (**B,C**) TLR7L (CL097), TLR9L (CpGA), zoledronate (Zol) alone or in combination (n=4). The phenotypic and functional features of γδ T cells, δ2+ and δ2- T-cell subsets were assessed through CD69, CD25 expression, IFNγ secretion and CD107 exposure upon further coculture with tumor cells. Bars represents mean±SEM. P-values calculated using the Wilcoxon matched pairs test with Bonferroni correction.

Supplementary Figure 5. The interplay between pDCs and γδ T cells require the activation of one partner

(**A-B**) Purified pDCs or γδ T cells from healthy donors' blood were cultured alone (white symbols) or in presence of the cross-talk partner (dark symbols) in absence of any stimulation. (**A**) The phenotypic and functional features of γδ T cells were assessed through CD69 and CD25 expression, IFNγ secretion and CD107 exposure upon further coculture with tumor cells (n=18 to 26). Bars represents mean±SEM. (**B**) The phenotypic and functional features of pDCs were assessed through CD40, CD80 and CD86 expression, TRAIL exposure and IFNα secretion (n=9 to 12). (**C**) IFNγ secretion during γδ T cell/pDC co-culture in absence of stimulation (-) or in presence of HMB-PP or Zol. Bars represents mean±SEM. P-values were calculated using the Wilcoxon matched pairs test.

Supplementary Figure 6. Membrane contacts and soluble factors are required for effective cross-talk between pDCs and δ2+ / δ2- T-cell subsets

Purified pDCs and γδ T cells from healthy donor' blood were cocultured together in the same well (white bars) or physically separated by a 0.4µm membrane (transwell, gray bars) in absence or presence of TLR7L (CL097), TLR9L (CpG_A) or zoledronate (Zol) as indicated. The phenotypic features (CD69 and CD25 expression) and cytotoxic properties (CD107 exposure upon coculture with tumor cells) of δ2+ and δ2- T-cell subsets were assessed to identify the requirement for membrane contacts and/or soluble factors for their cross-talk with pDCs (n=8). Bars represents mean±SEM. P-values were calculated using the Wilcoxon matched pairs test.

Supplementary Figure 7. Experimental schemes for blocking experiments

(**A**) Strategy for blocking single molecules, BTN3A or mixtures to determine the mechanism of the pDC- γδ T cells cross-talk. (**B**) Strategy for blocking single molecules, BTN3A or mixtures to determine the mechanism of the γδ T cell-pDC cross-talk.

Supplementary Figure 8. pDCs and γδ T cells differentially expressed TNFαRI, TNFαRII and IFNαRI/RII

(A) Basal expression of TNFαRI, TNFαRII, IFNαRI and IFNαRII by γδ T cells and pDCs determined within PBMC (n=4). (**B**) The expression of TNFαRI, TNFαRII, IFNαRI and IFNαRII by γδ T cells and pDCs was evaluated during the coculture of purified pDCs and γδ T cells in presence or not of HMB-PP, CLO97, CpGA or Zol (n=2).

Supplementary Figure 9. The blocking of BTN3A or the use of the Supermix doesn't prevent pDC response to TLR7/9L stimulation

Purified pDCs from healthy donor' blood were cultured with or without TLR7L (CL097), TLR9L (CpG_A) or zoledronate (Zol) in presence or not of blocking antibodies. The ability of pDCs to upregulate activation molecules (CD40, CD80, CD86), express TRAIL and secrete IFNα was then evaluated. (**A**) Cultures performed in presence or not of anti-BTN3A blocking antibody (n=6). (**B**) Cultures performed in presence or not of the Supermix composed of anti-IFNAR, -TNFR1, -TNFR2 and -OX40 blocking antibodies together with anti-BTN3A1 blocking antibodies (n=6). Bars represents mean±SEM. P-values were calculated using the Wilcoxon matched pairs test.

Supplementary Figure 10. Relative impact of BTN3A compared to the other molecules targeted by the Supermix on the pDCs-γδ T cells cross talk

Purified pDCs from healthy donors' blood were pre-incubated either with single anti-BTN3A antibodies (n=12) or a Supermix composed of anti-IFNAR2, -TNFR1/TNFR2 and -OX40 antibodies together with anti-BTN3A blocking antibody (n=12), and cocultured with purified γδT cells in the presence or not of TLR7-L (CL097) (light gray bars), TLR9-L (CpG_A) (dark gray bars) or zoledronate (Zol) (black bars). The features of γδT cells were then depicted: the activation status (CD25 and CD69 expression) and the cytotoxic activity (CD107 surface exposure) were analyzed by flow cytometry; IFNγ secretion was measured by CBA in the supernatants. Bars represents mean±SEM. P-values were calculated using the Wilcoxon-matched pairs test (full lines) or Mann-Whitney test (dotted lines) (*p<0.05,**p<0.01,***p<0.001).

Supplementary Figure 11. γδ T cells keep their ability to respond to HMB-PP stimulation in presence of blocking antibodies composing the "mix -" or blocking of BTN3A
(**A**) Purified γδ T cells from healthy donor' blood were cultured with or without HMB-PP or zoledronate (Zol) in presence or not of the "mix -" composed of anti-IFNγR, -NKp30 and -GITR blocking antibodies (n=4-10). The ability to upregulate activation molecules (CD69, CD25) and secrete IFNγ was then evaluated on γδ T cells. (**B**) Purified γδ T cells from healthy donor' blood were pre-activated for 4 hours with or without HMB-PP or zoledronate (Zol) and mixed with purified pDCs pre-cultured for 2 hours in presence or not of anti-BTN3A blocking antibodies (n=8). The ability to upregulate activation molecules (CD69, CD25) and secrete IFNγ was then evaluated on γδ T cells. P-values were calculated using the Wilcoxon matched pairs test.

Supplementary Figure 12. Relative impact of BTN3A compared to the other molecules targeted by the Supermix on the γδ T cells-pDCs cross talk
Purified γδT cells from healthy donor' blood were pre-activated with HMB-PP (light gray symbols) or Zol (dark gray symbols) during 4hours in absence (condition "BTN3A", n=8) or presence of a mixture of blocking antibodies composed of anti-IFNγR, -NKp30, and -GITR antibodies (condition "Supermix", n=9-10), and mixed with pDCs pre-blocked with single anti-BTN3A for 2hours. The features of pDCs were then depicted: the activation status (MFI CD40 on CD40-positive cells) and cytotoxic properties (TRAIL) were analyzed by flow cytometry, whereas the cytokine secretion (IFNα and IP10) was assessed by CBA in the supernatants. P-values were calculated using the Wilcoxon-matched pairs test (full lines) or Mann-Whitney test (dotted lines) (*p<0.05,**p<0.01,***p<0.001).

Supplementary Figure 1

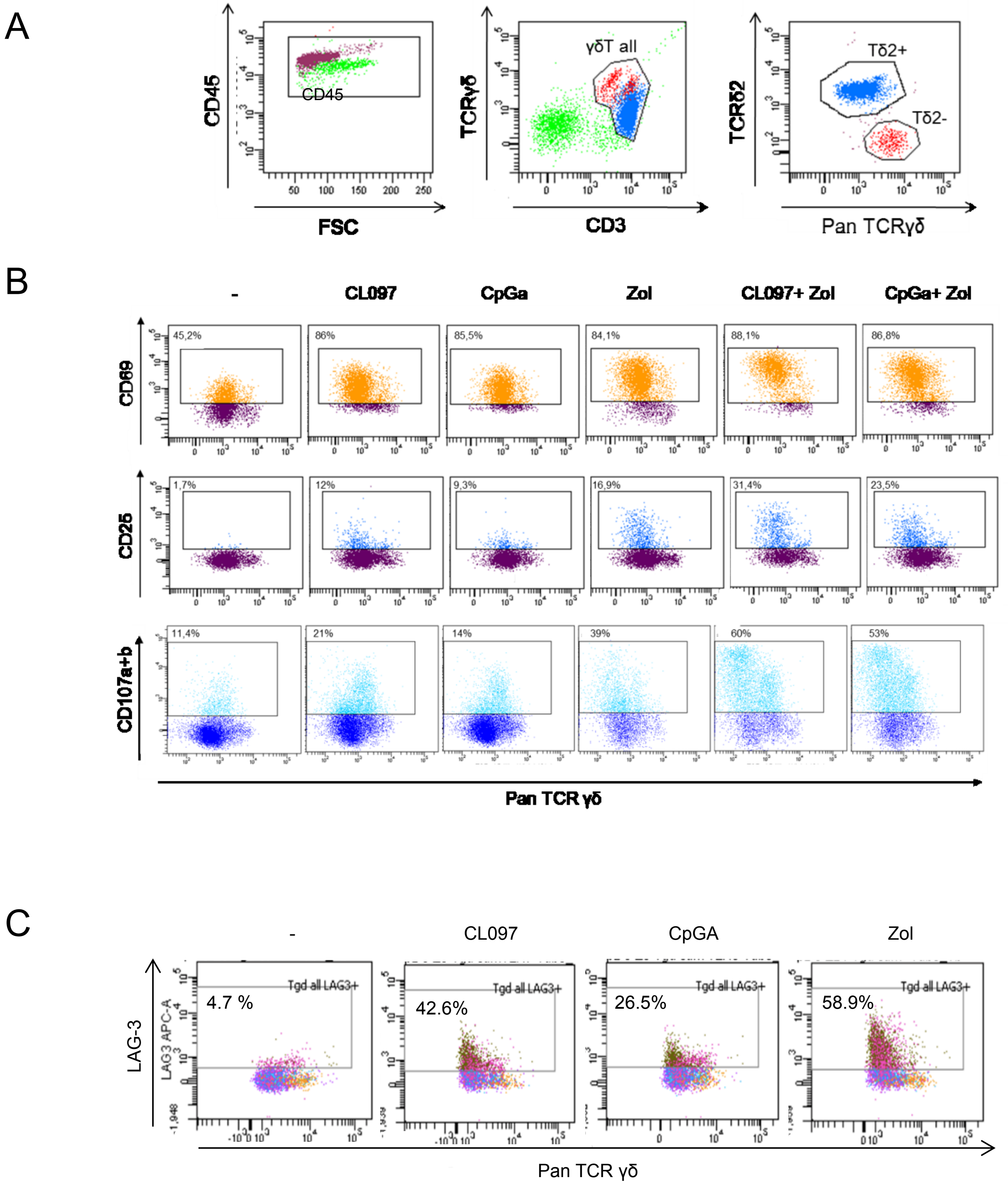

Supplementary Figure 1 follow

D

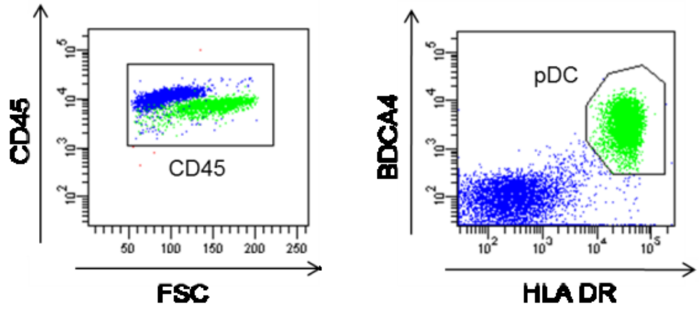

E

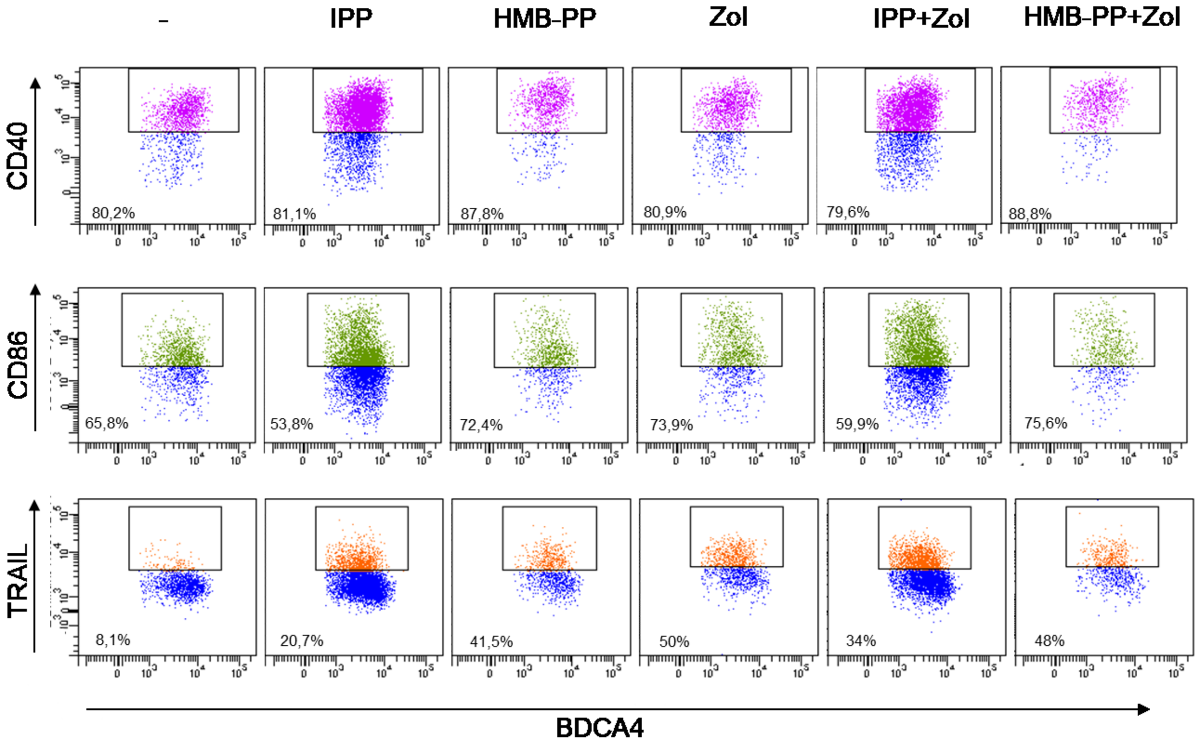

F

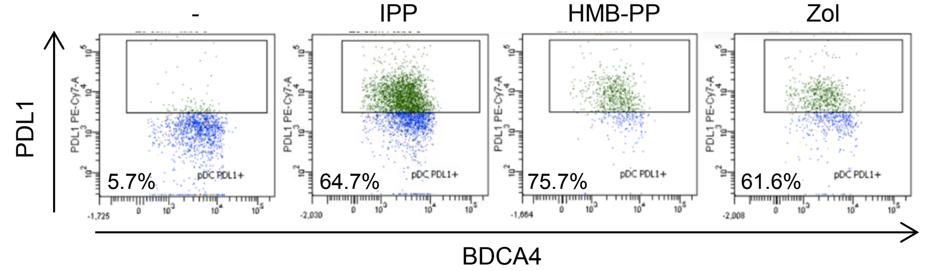

Supplementary Figure 2

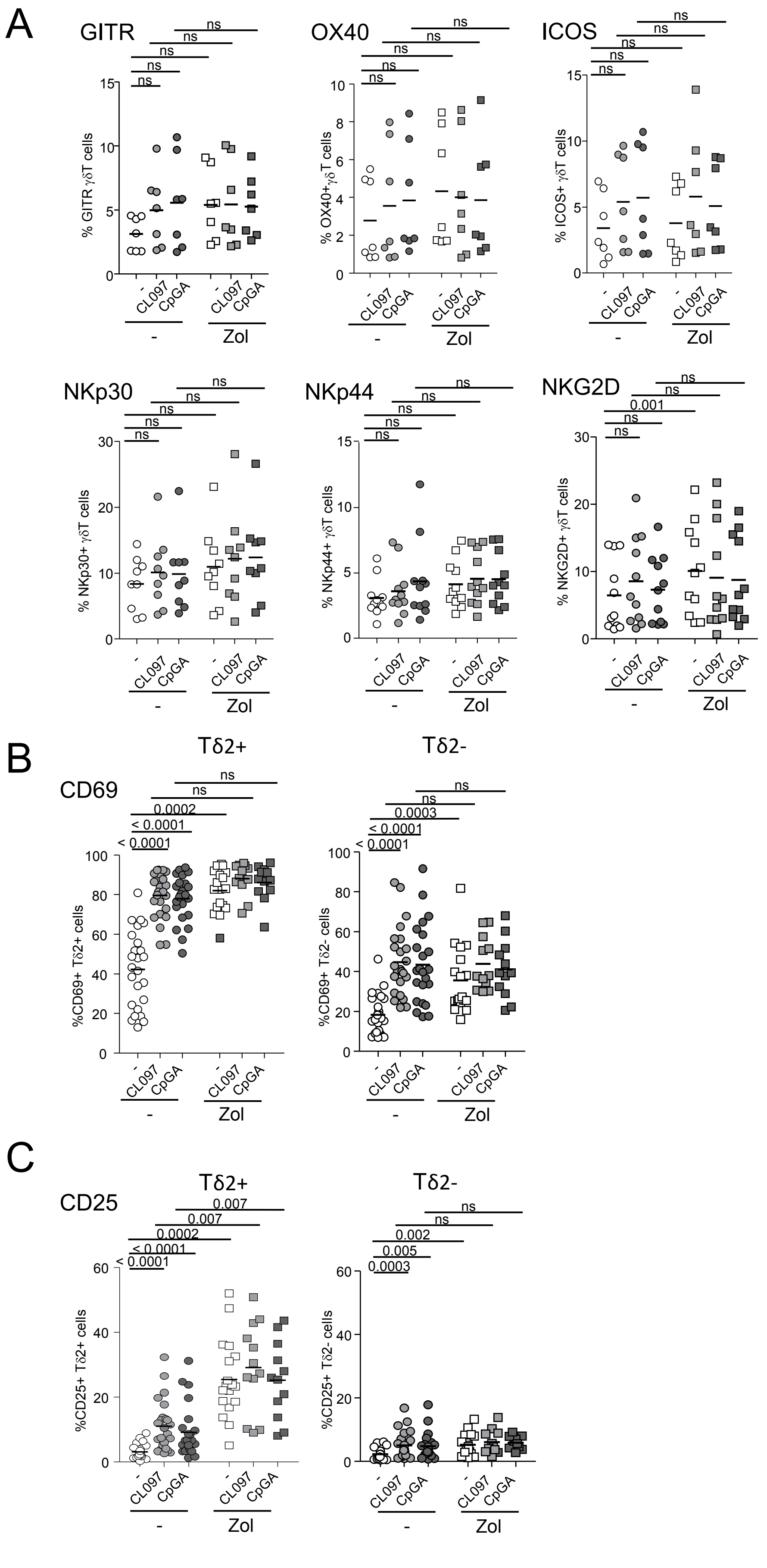

Supplementary Figure 2 follow

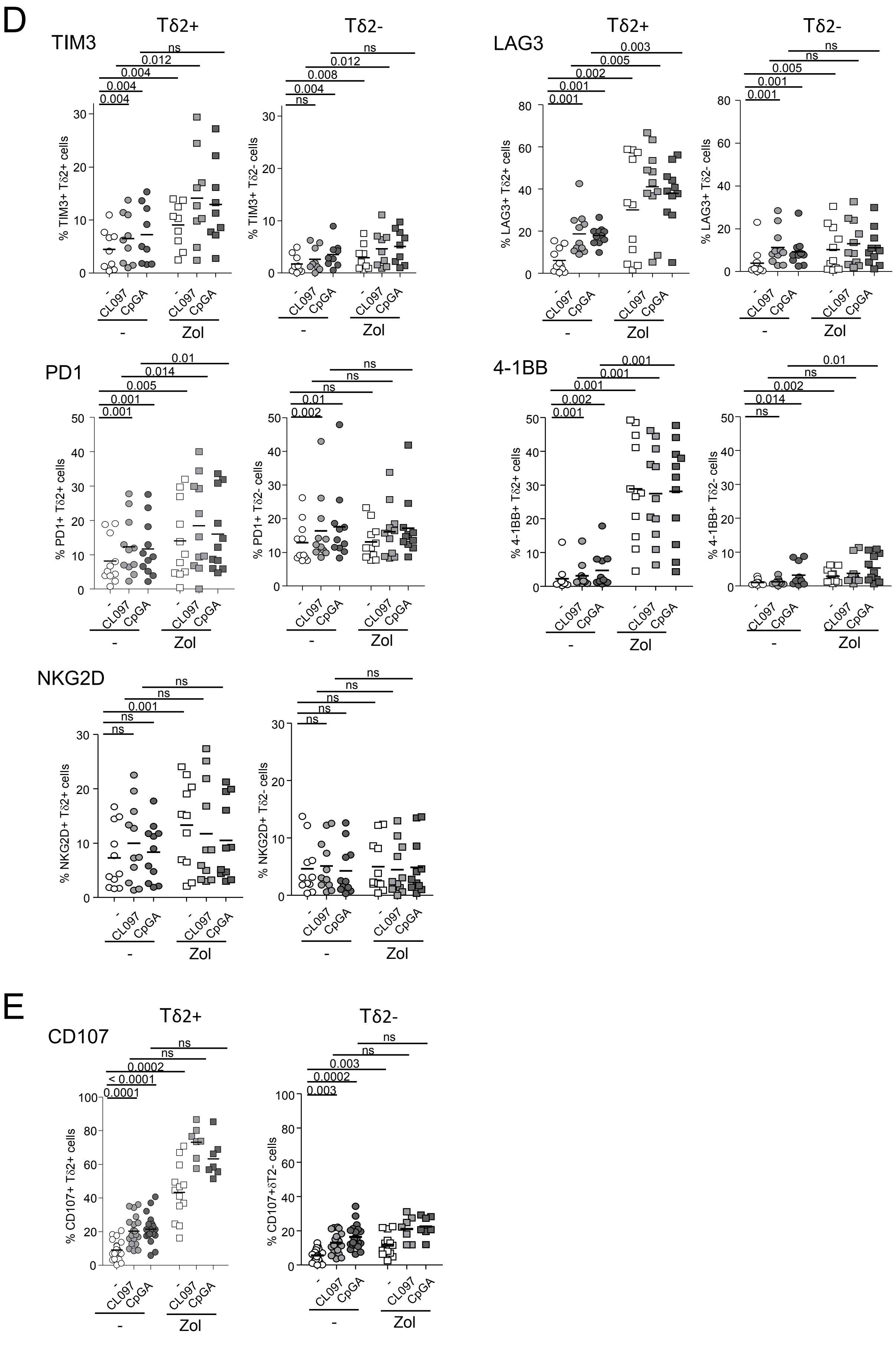

Supplementary Figure 3

A

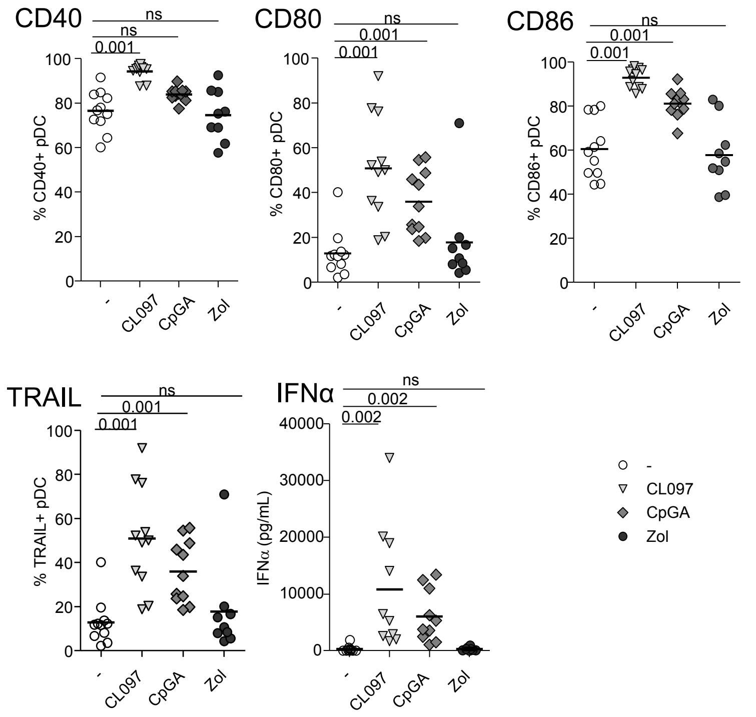

B

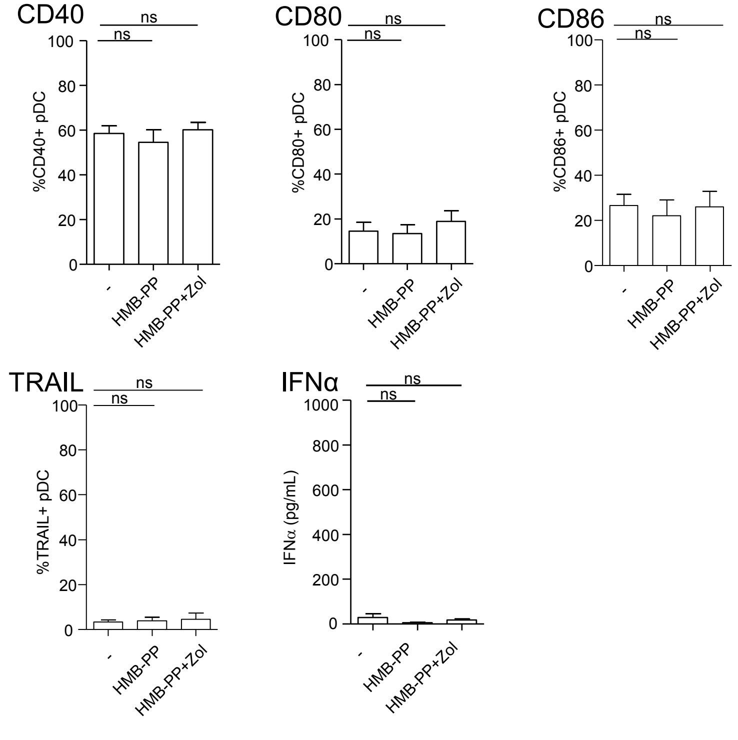

Supplementary Figure 4

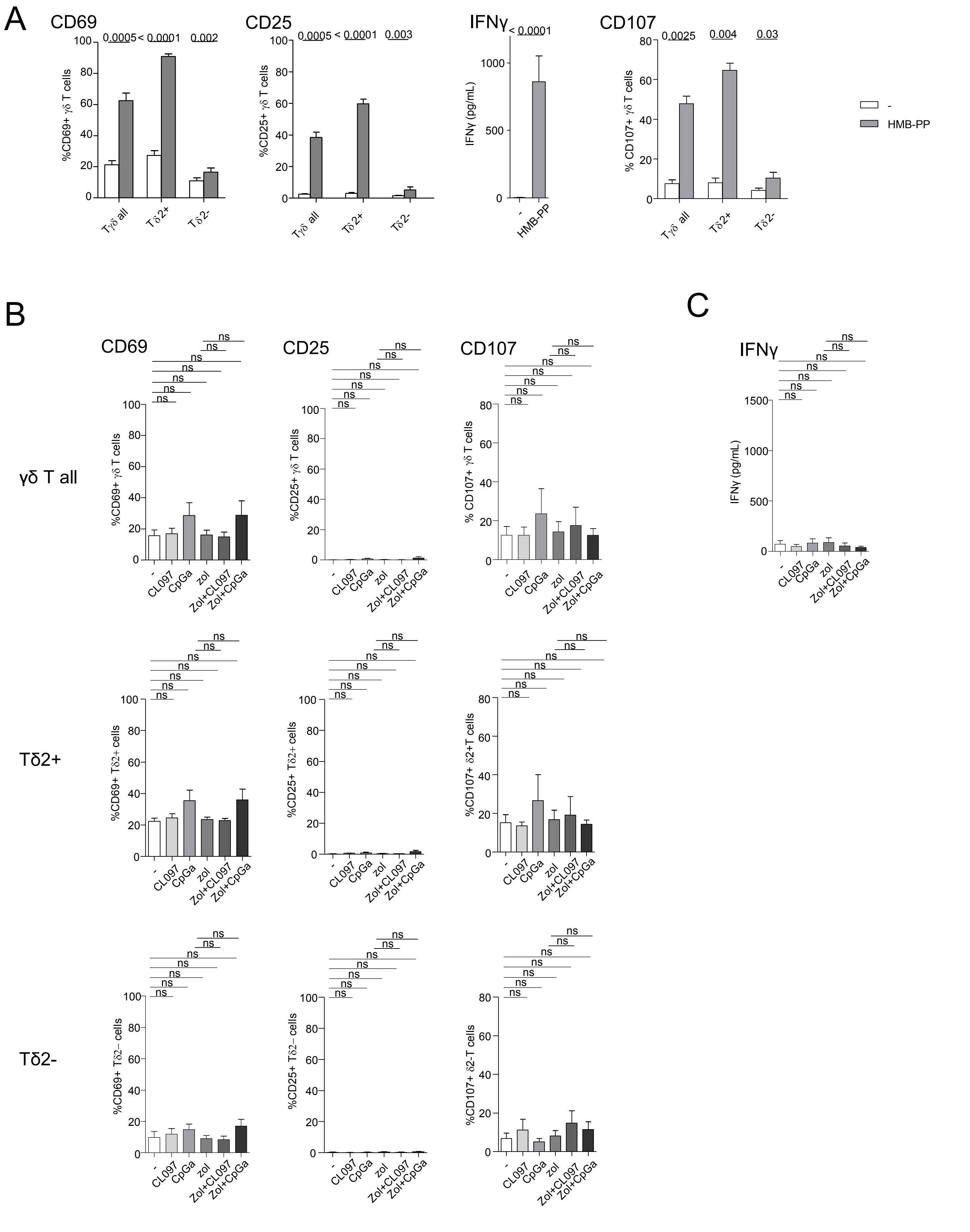

Supplementary Figure 5

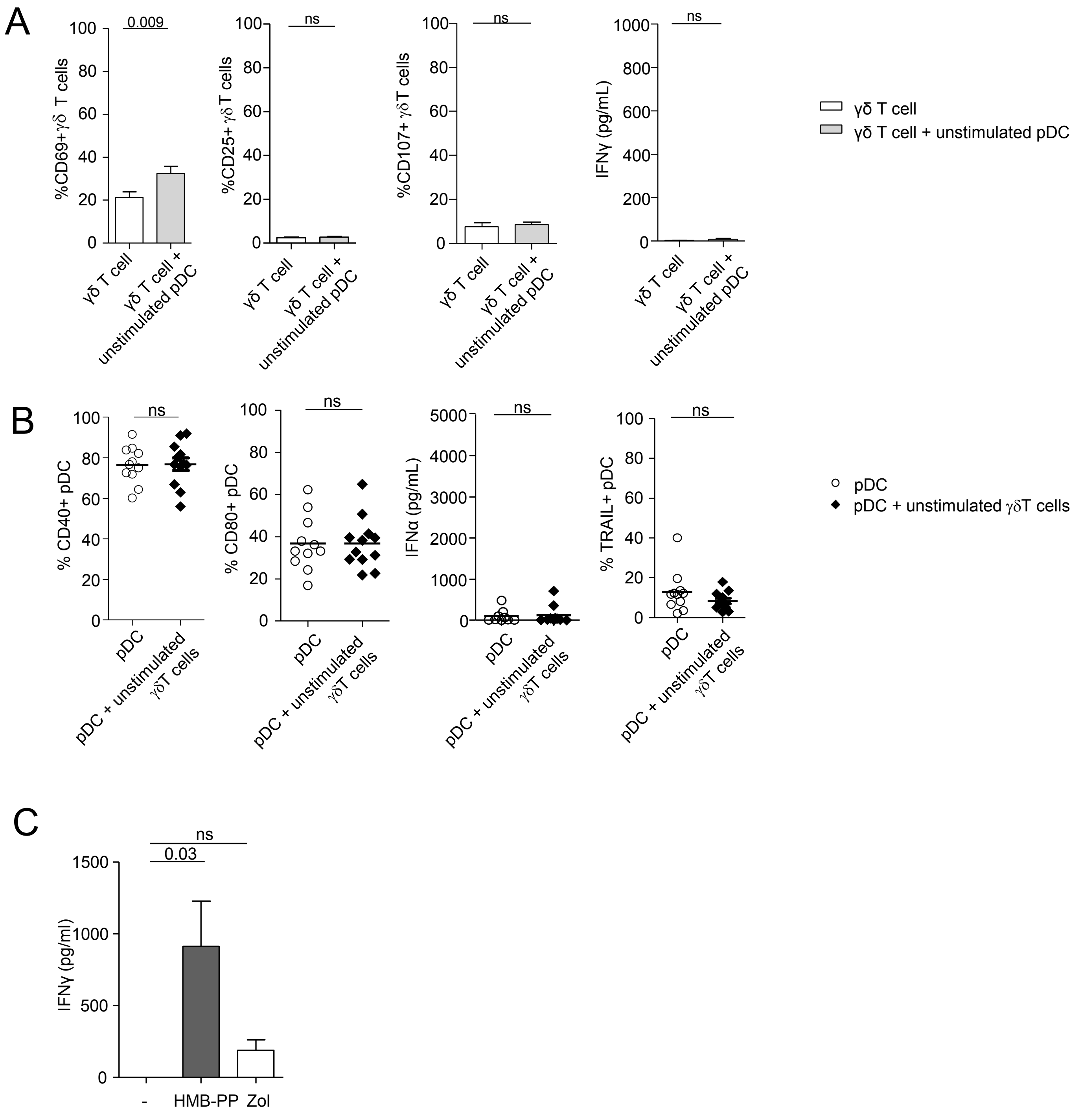

Supplementary Figure 6

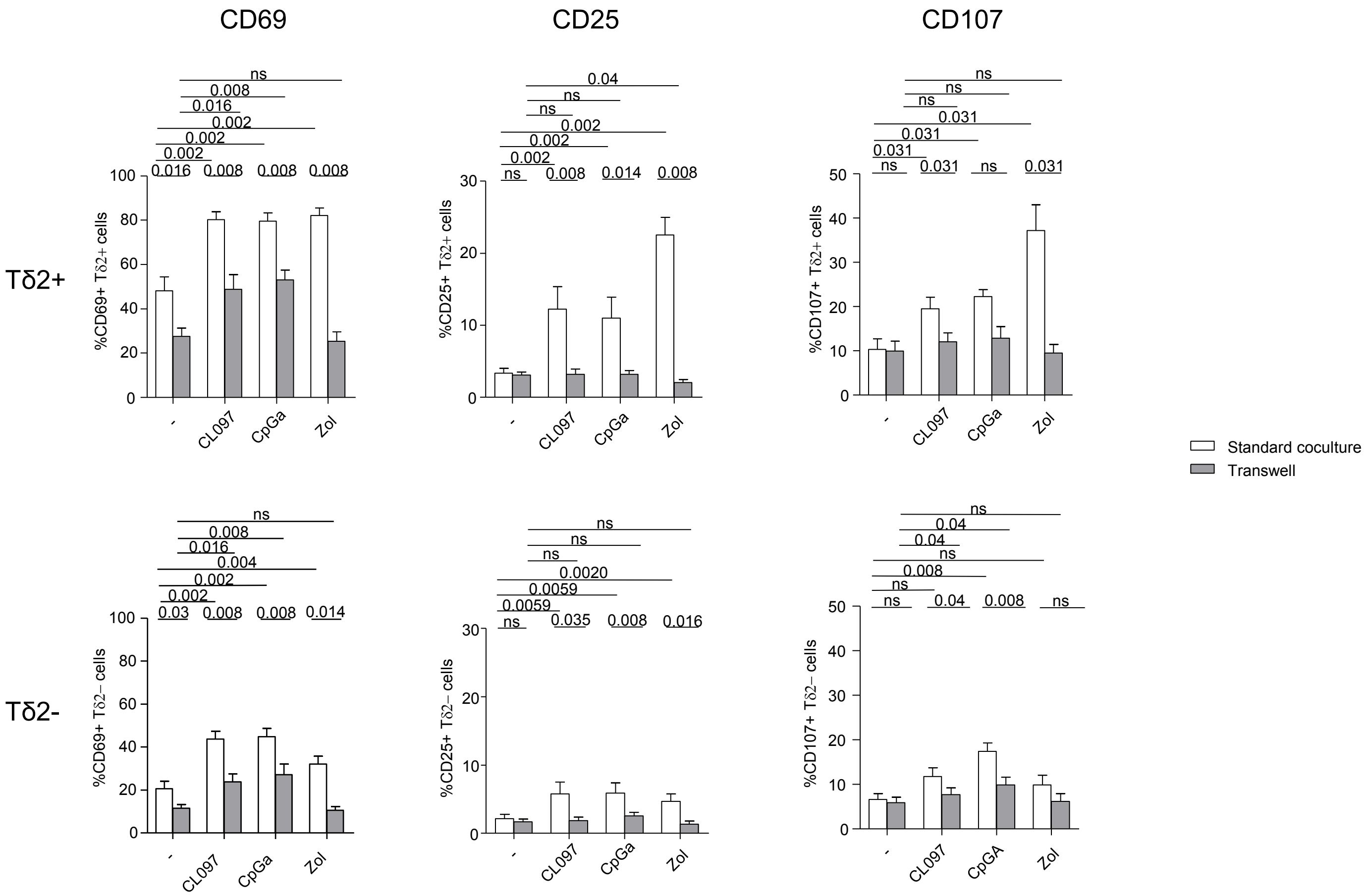

Supplementary Figure 7

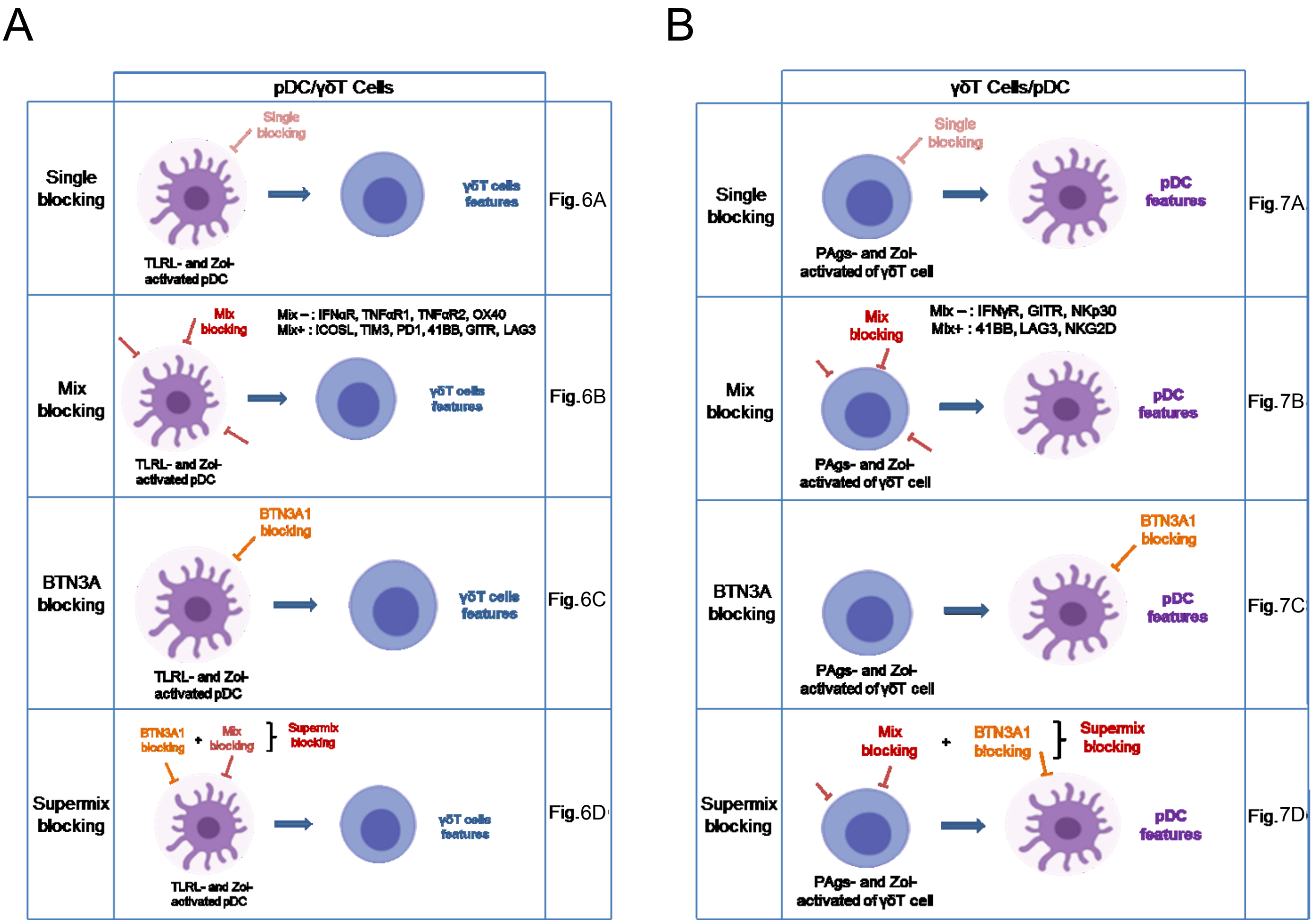

Supplementary Figure 8

A

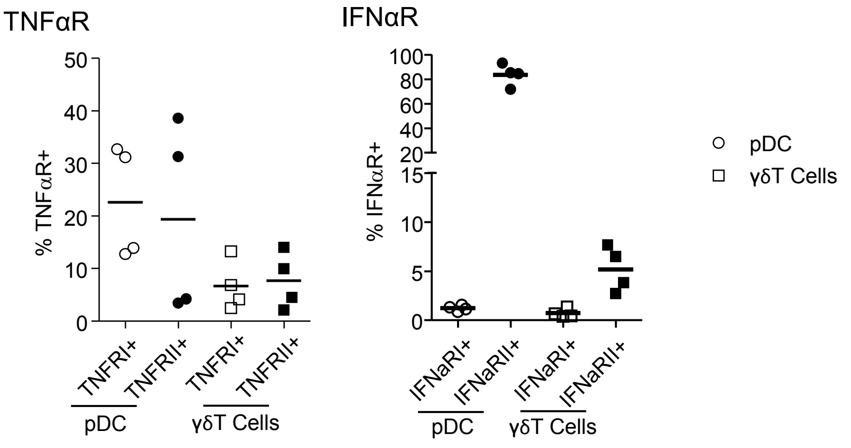

B

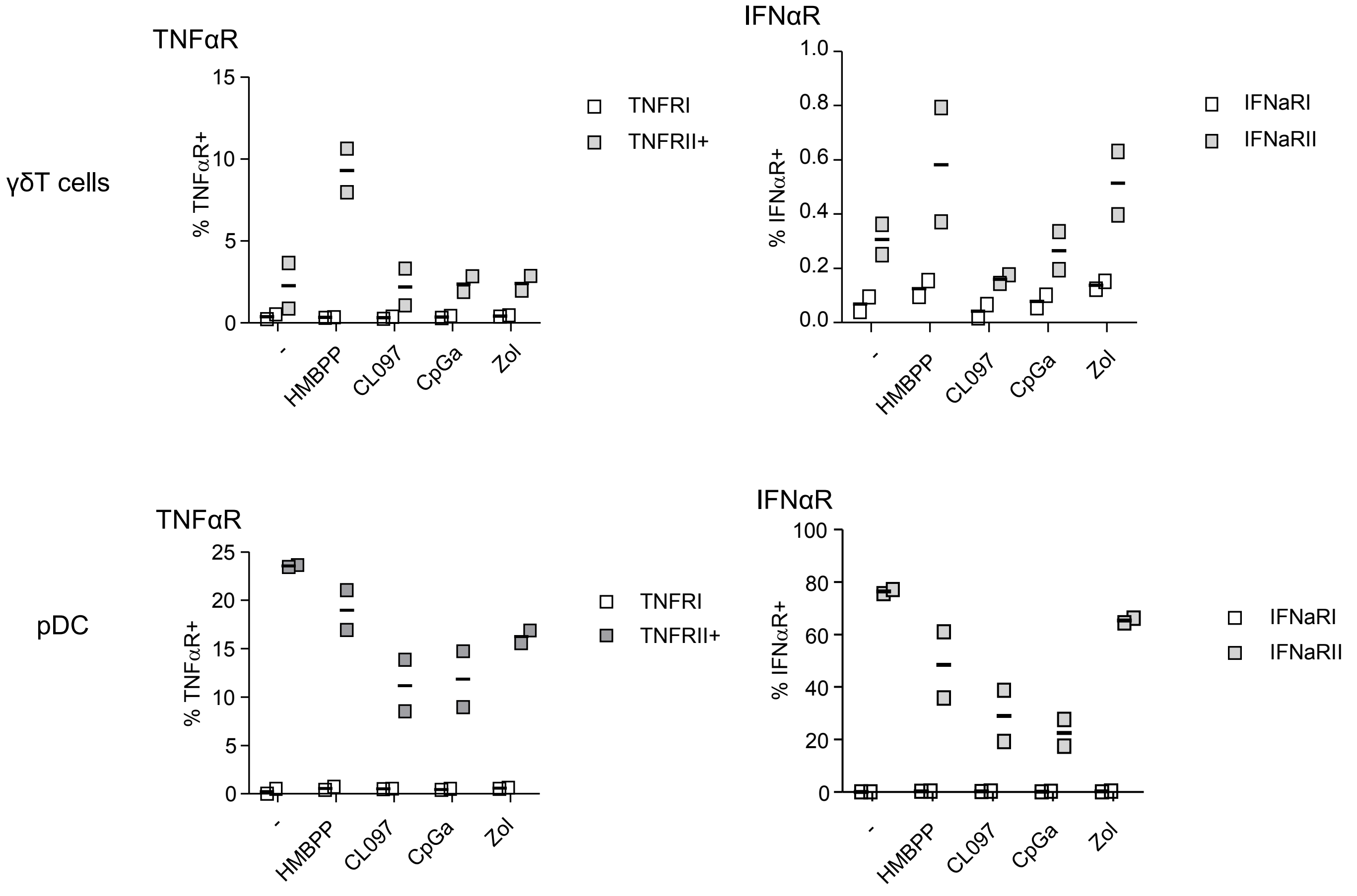

Supplementary Figure 9

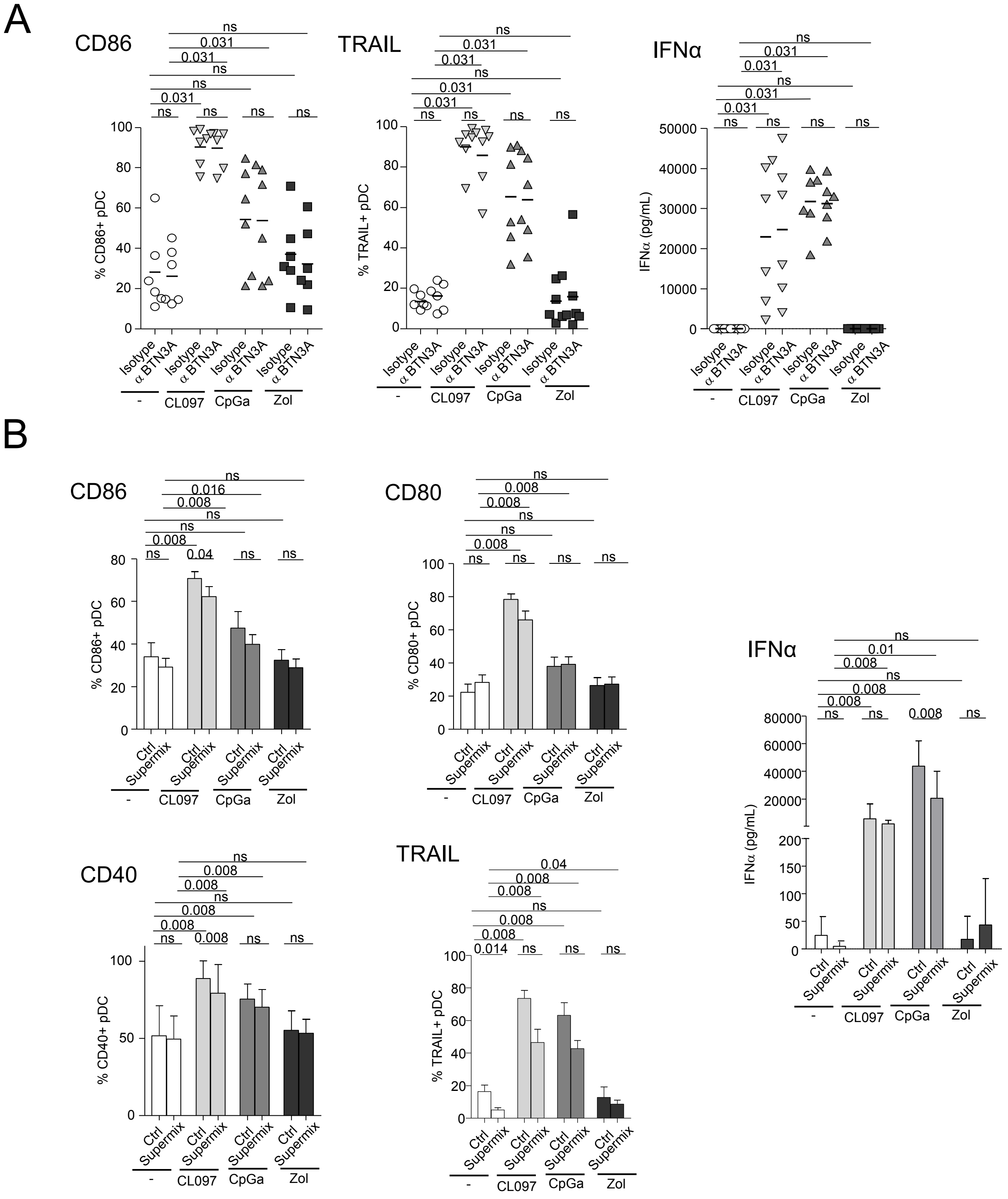

Supplementary Figure 10

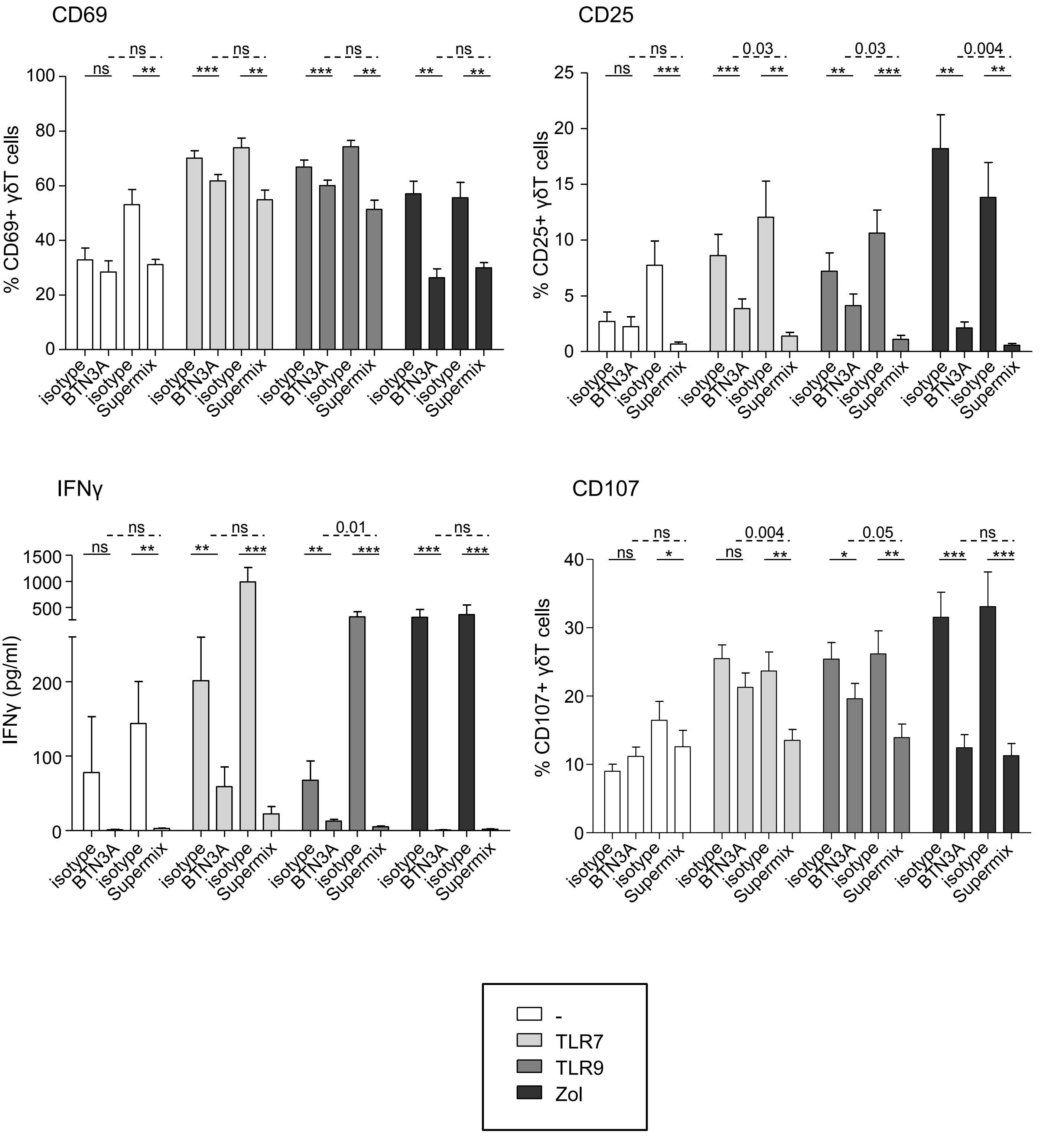

Supplementary Figure 11

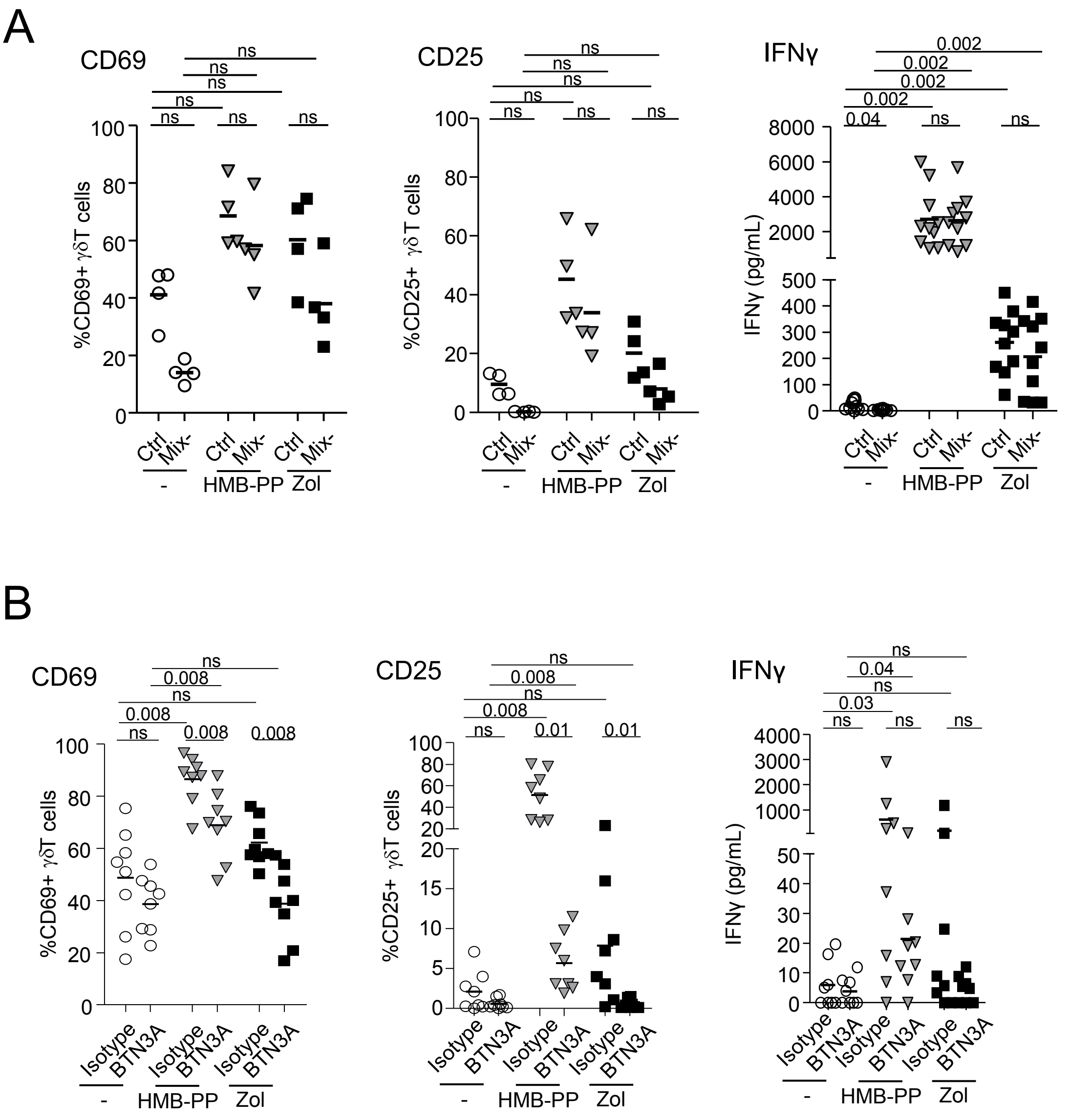

Supplementary Figure 12

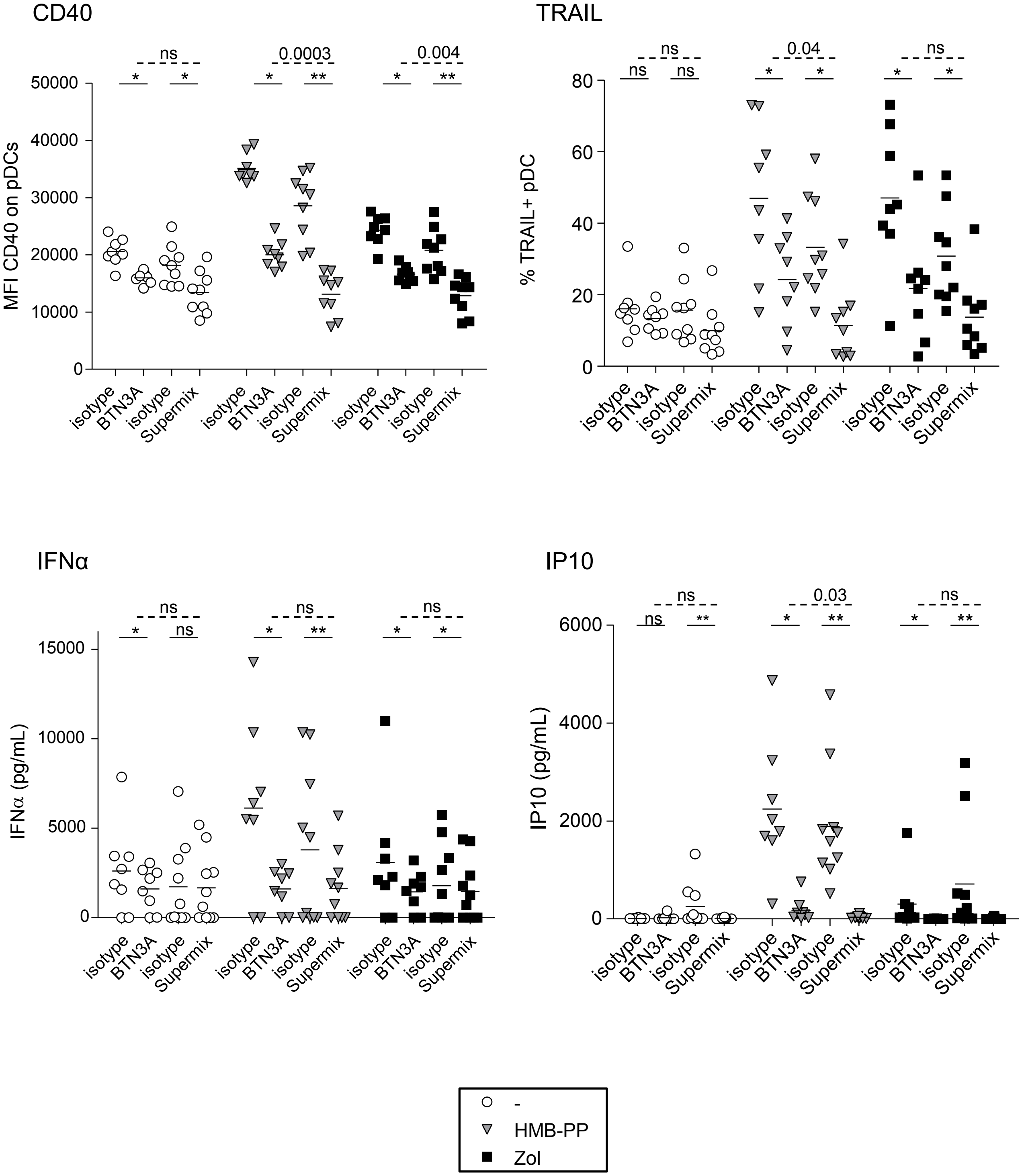

Supplement: Supplementary file 1 [file Data_Sheet_1.pdf]
